# The Causative Gene in Chanarian Dorfman Syndrome Regulates Lipid Droplet Homeostasis in *C*. *elegans*

**DOI:** 10.1371/journal.pgen.1005284

**Published:** 2015-06-17

**Authors:** Meng Xie, Richard Roy

**Affiliations:** Department of Biology, McGill University, Penfield, Montreal, Quebec, Canada; University of California San Francisco, UNITED STATES

## Abstract

AMP-activated kinase (AMPK) is a key regulator of many cellular mechanisms required for adjustment to various stresses induced by the changing environment. In *C*. *elegans* dauer larvae AMPK-null mutants expire prematurely due to hyperactive Adipose Triglyceride Lipase (ATGL-1) followed by rapid depletion of triglyceride stores. We found that the compromise of one of the three *C*. *elegans* orthologues of human *cgi-58* significantly improves the survival of AMPK-deficient dauers. We also provide evidence that *C*. *elegans* CGI-58 acts as a co-activator of ATGL-1, while it also functions cooperatively to maintain regular lipid droplet structure. Surprisingly, we show that it also acts independently of ATGL-1 to restrict lipid droplet coalescence by altering the surface abundance and composition of long chain (C20) polyunsaturated fatty acids (PUFAs). Our data reveal a novel structural role of CGI-58 in maintaining lipid droplet homeostasis through its effects on droplet composition, morphology and lipid hydrolysis; a conserved function that may account for some of the ATGL-1-independent features unique to Chanarin-Dorfman Syndrome.

## Author Summary

Chanarin-Dorfman Syndrome (CDS) is a rare metabolic disease characterized by an abnormal accumulation of lipids in various tissues and organs due to a failure in lipid breakdown. Characteristic clinical features exhibited by affected patients include scaly skin (ichthyosis), enlarged liver, blurred vision among others. CDS is caused by mutation of the *cgi-58* gene, which is essential for lipid breakdown, but may also have additional cellular functions. Here, we demonstrate that in *C*. *elegans* CGI-58 acts both as a key player in lipid breakdown, but it is also required to maintain the barrier that defines the size, shape and catalytic efficacy of the major lipid storage site-the lipid droplets. We provide a genetically tractable animal model of CDS that reproduces many of the defects observed in affected CDS individuals.

## Introduction

Most organisms possess a remarkable capacity to sense environmental variation and to modify their physiology accordingly to adapt to such changes. During periods of nutrient scarcity, animals prioritize the use of limiting macromolecules for survival benefit, even if it results in a temporary suspension of reproductive development [1–5]. Similarly, in the free-living nematode *C*. *elegans*, a highly resistant “dauer” stage provides an alternative developmental pathway that increases the animals’ fitness upon encountering suboptimal growth conditions such as resource depletion or increases in either population density or growing temperature.

In *C*. *elegans*, parallel cross-talk among several genetic pathways determines an “all or none” dauer entry response. This developmental decision is dictated by neuronal sensing of environmental indicators followed by signal transduction in neuroendocrine cells to eventually converge on a nuclear hormone receptor-mediated transcriptional cascade [6].

One of the common targets of these three genetic pathways is the AMP-activated protein kinase (AMPK) [7]. Upon activation by its upstream activating protein kinase LKB1/PAR-4, AMPK will in turn phosphorylate downstream targets to promote catabolic processes while simultaneously blocking anabolic processes to restore energy homeostasis [8–10].

During normal growth conditions nutrient/energy is initially stored in the form of glycogen, while surplus calories are packaged into triglyceride molecules for use during situations when energy demands exceed nutrient input. The triglycerides are stockpiled in monolayer phospholipid-encapsulated organelles called lipid droplets, which serve as the major triglyceride reserves in all metazoans. Their energy-rich contents can be accessed in a regulated manner based on metabolic need.

In situations of intense energy demand, triglycerides are hydrolyzed to free fatty acids (FFAs) in a series of sequential reactions that are catalyzed by substrate-specific lipase enzymes that each release a single FFA. These lipases include adipose triglyceride lipase (ATGL), hormone-sensitive lipase (HSL) and monoglyceride lipase (MGL). Observation of enlarged fat deposits and triglyceride accumulation within multiple tissues in ATGL-deficient mice suggest that ATGL catalyzes the initial rate-limiting step of triglyceride breakdown to release FFA and diacylglyceride (DG) [11]. The latter is subsequently cleaved by HSL and MGL to generate glycerol and FFAs, and the final products diffuse from the adipose tissue into the circulation.

Although ATGL possesses significant catalytic activity in vitro it is unlikely to function alone in vivo. Characterized by its presence on intracellular lipid droplets in most tissues, CGI-58 (comparative gene identification-58) is one of the major lipid droplet-associated proteins identified in mammals and is the causative gene for human Chanarin-Dorfman syndrome (CDS) [12]. Recent studies have revealed that CGI-58 acts as a co-activator for ATGL in mammals, by binding directly to, and enhancing the hydrolase activity and broadening the substrate specificity of ATGL [13], [14]. The interaction between CGI-58 and ATGL is necessary but not sufficient for activation of the latter, while the ability to interact with ATGL is insufficient to recruit CGI-58 to the lipid droplet. Interaction with the lipid droplet may therefore require other proteins or regulatory mechanisms [15].

During the dauer stage in *C*. *elegans* AMPK blocks the rapid hydrolysis of the accumulated triglyceride stockpiles through the phospho-inhibition of ATGL-1 leading to the early expiration of the mutant animals [16]. We show here that the removal of the *C*. *elegans* homologue of the human CGI-58 protein improves the survival and many of the defects associated with compromised AMPK function by tethering ATGL-1 to the lipid droplets and increasing its lipase activity. In addition, we provide evidence that CGI-58 plays an additional role independent of ATGL in maintaining lipid droplet homeostasis in both *C*. *elegans* and in mammalian cells, the compromise of which leads to increased lipid coalescence which may account for the lipid aggregates seen in CDS patients. Our findings suggest that CGI-58 not only recruits and tethers ATGL-1 onto its substrate, but also functions independently of ATGL-1 to establish and maintain a barrier surrounding the lipid droplet to limit droplet coalescence. By altering the abundance of C20 polyunsaturated fatty acid (PUFA) and Phosphatidic acid (PA) on the droplet surface CGI-58 regulates lipid droplet morphology while simulataneously optimizing droplet size and ATGL-1-dependent lipolysis.

## Results

### Reducing the Levels of the *C*. *elegans* CGI-58 Homologue Enhances the Survival of AMPK-Deficient Dauer Larvae

**Fig 1:**
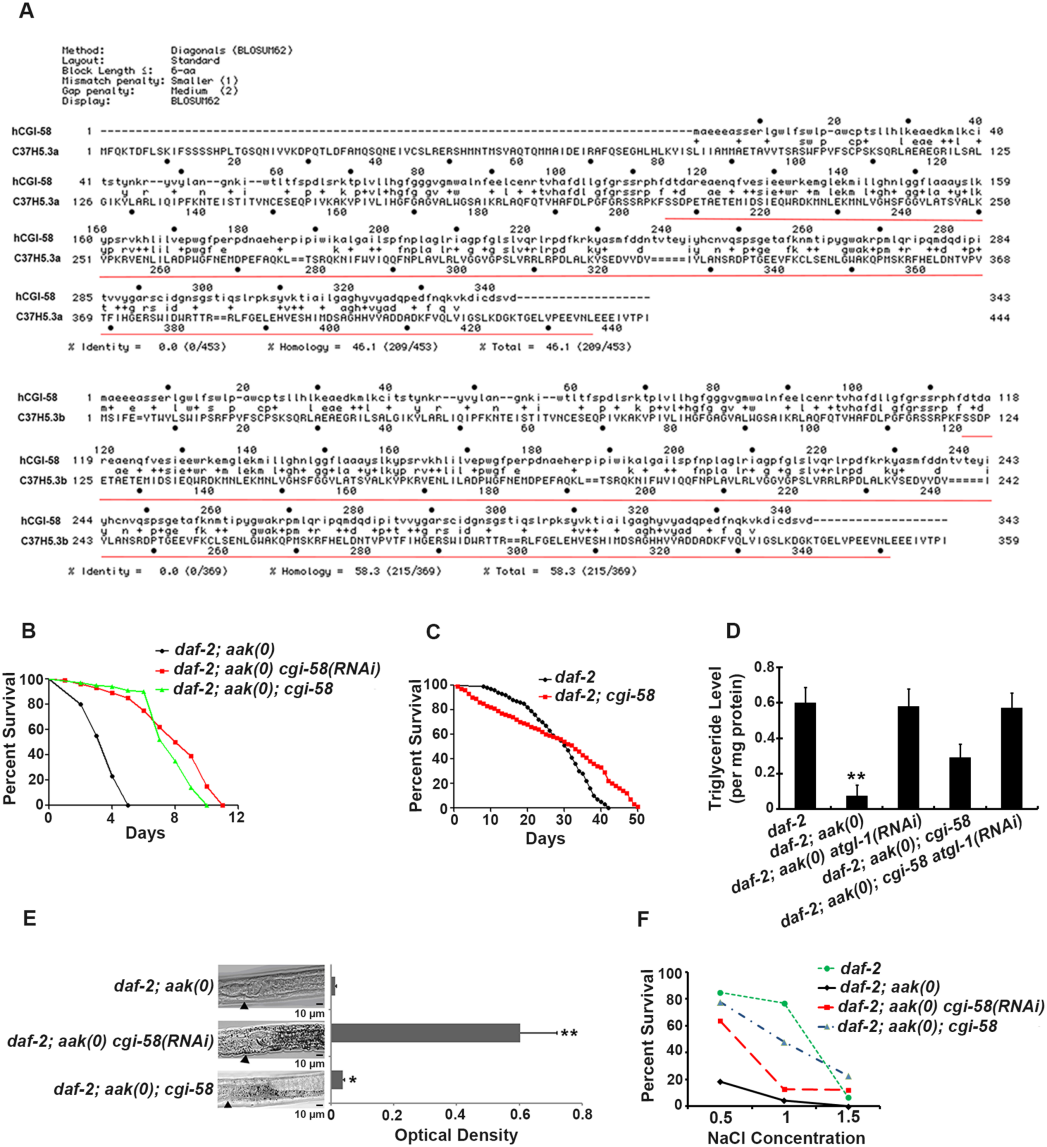
Elimination of *cgi-58* rescued both triglyceride levels and excretory defects in *daf-2; aak(0)* mutant Dauer larvae. **(A)** Protein alignment of the two isoforms of *C*. *elegans* CGI-58 (C37H5.a and C37H5.b) with human CGI-58. The red underlined amino acids indicate the deleted region in the *ok3245* allele. **(B)** Elimination of *cgi-58* by RNAi feeding or by using the *ok3245* allele significantly enhanced the survival of *daf-2; aak-1; aak-2* mutant dauer larvae. *aak-1; aak-2* is represented as *aak(0)* in all Figs. A Log-rank (Mantel-Cox) test was used to statistically compare the survival curves in all Figs. **(C)** Mutation of *cgi-58* in control *daf-2* animals significantly enhanced their survival during dauer stage (P = 0.0003). **(D)** *cgi-58* protects the triglyceride stores from depletion in *daf-2; aak(0)* mutant dauer larvae. Colorimetric analysis of triglyceride content in day 4 dauer larvae. ** indicates statistical significance (P<0.01) compared to all four of the other genotypes using one-way ANOVA followed by a Tukey HSD test. Error bars indicate SD of three independent experiments. The same statistical analysis was applied for all subsequent experiments performed hereafter. **(E)** Elimination of *cgi-58* protects the triglyceride stockpile in *daf-2; aak(0)* mutant dauer larvae. Oil Red O staining of day 4 dauer larvae. Arrowhead indicates the junction between the pharynx (left) and the intestine (right). Oil Red O staining intensity was evaluated by measuring optical density. * and ** indicate statistical significance (P<0.05 and P<0.01, respectively) compared to *daf-2; aak(0)* mutant dauer larvae. Error bars indicate SD of 20 animals. Scale bar = 10 μm. **(F)** Osmoregulatory defects typical of AMPK dauer larvae were corrected by reducing *cgi-58* function. *cgi-58* compromise restores osmoresistance of day 4 *daf-2; aak(0)* mutant dauer larvae following culture in varying NaCl concentrations for 24 hours at 25°C. The data represent a pool of three independent experiments. Survival of *daf-2*, *daf-2; aak(0) cgi-58(RNAi)* and *daf-2; aak(0); cgi-58* dauers is significantly higher than *daf-2; aak(0)* dauers at concentrations of both 1M and 1.5M NaCl, analyzed using one-way ANOVA followed by a Tukey HSD test.

Although AMPK negatively regulates ATGL-1 during the dauer stage, we and others have identified factors that are required for, or enhance ATGL-1 activity [15], [17], [18]. In mammalian cells ATGL is regulated by a complex relay mechanism between lipid droplet-associated proteins such as the Perilipins, G0S2 and CGI-58. At present, no clear orthologue of G0S2, or the Perilipin protein family has been identified in the annotation of the *C*. *elegans* genome sequence. To explore how ATGL-1 may be regulated during the dauer stage, we characterized the function of the *C*. *elegans* orthologue of mammalian CGI-58, which we identified in a genome-wide survey for genes that phenocopied *atgl-1(RNAi)* by enhancing dauer survival of animals with mutations in both isoforms of their AMPK α subunit (*aak-1* and *aak-2*, which will be presented as *aak(0)* throughout the study) [17]. The predicted *C*. *elegans* gene C37H5.3 is expressed as two alternative transcripts, both of which encode a protein that is 46.1% and 58.3% similar to the human CGI-58 protein, respectively (Fig 1A). Importantly, one of the N-terminal tryptophan residues previously shown to be critical for the correct localization and ATGL-activating function of mammalian CGI-58 is conserved in *C*. *elegans* [15]. There are two other genes in the *C*. *elegans* genome that share sequence homology with the mammalian CGI-58, *lid-1* and C37H5.2, where the former has recently been shown to regulate ATGL-1-dependant lipolysis during fasting in *C*. *elegans* [19]. However, neither of these two genes were recovered from our genome-wide RNAi survey [17]. Furthermore, in our directed RNAi assays, elimination of either *lid-1* or C37H5.2 did not enhance dauer survival in insulin-signalling compromised *(daf-2)* dauer larvae that lacked AMPK function (S1 Fig). For this reason we chose to further investigate C37H5.3 function during dauer development and will be referred to hereafter *as cgi-58* throughout our study.

Using both RNAi and an available *cgi-58* mutant with a ~800bp deletion that eliminates most of the C-terminal part of the protein (*ok3245*) (underlined in red in Fig 1A), we found that elimination of *C*. *elegans* CGI-58 protein significantly increased the survival of AMPK mutant dauer larvae (Fig 1B). Moreover, the maximal survival of control *daf-2* dauers was also enhanced in larvae that lacked *cgi-58*, despite a lower mean survival rate within the initial 30 days (Fig 1C). In addition, removal of *C*. *elegans* CGI-58 increased the total triglyceride levels as measured by both colorimetric (Fig 1D) and Oil Red O staining (Fig 1E) methods, and improved their osmoresistance when cultured in high salt conditions at dauer day 4 stage (Fig 1F); all of which are characteristic defects observed in AMPK mutant dauer larvae. Taken together, we conclude that the depletion of *C*. *elegans* CGI-58 can prolong the survival of AMPK mutant dauer larvae by conserving lipid reserves and thereby indirectly improve excretory/osmoregulatory function, probably through maintenance of organismal energy levels.

### CGI-58 Physically Interacts with ATGL-1 In Vivo and Affects ATGL-1 Localization on Lipid Droplets

**Fig 2:**
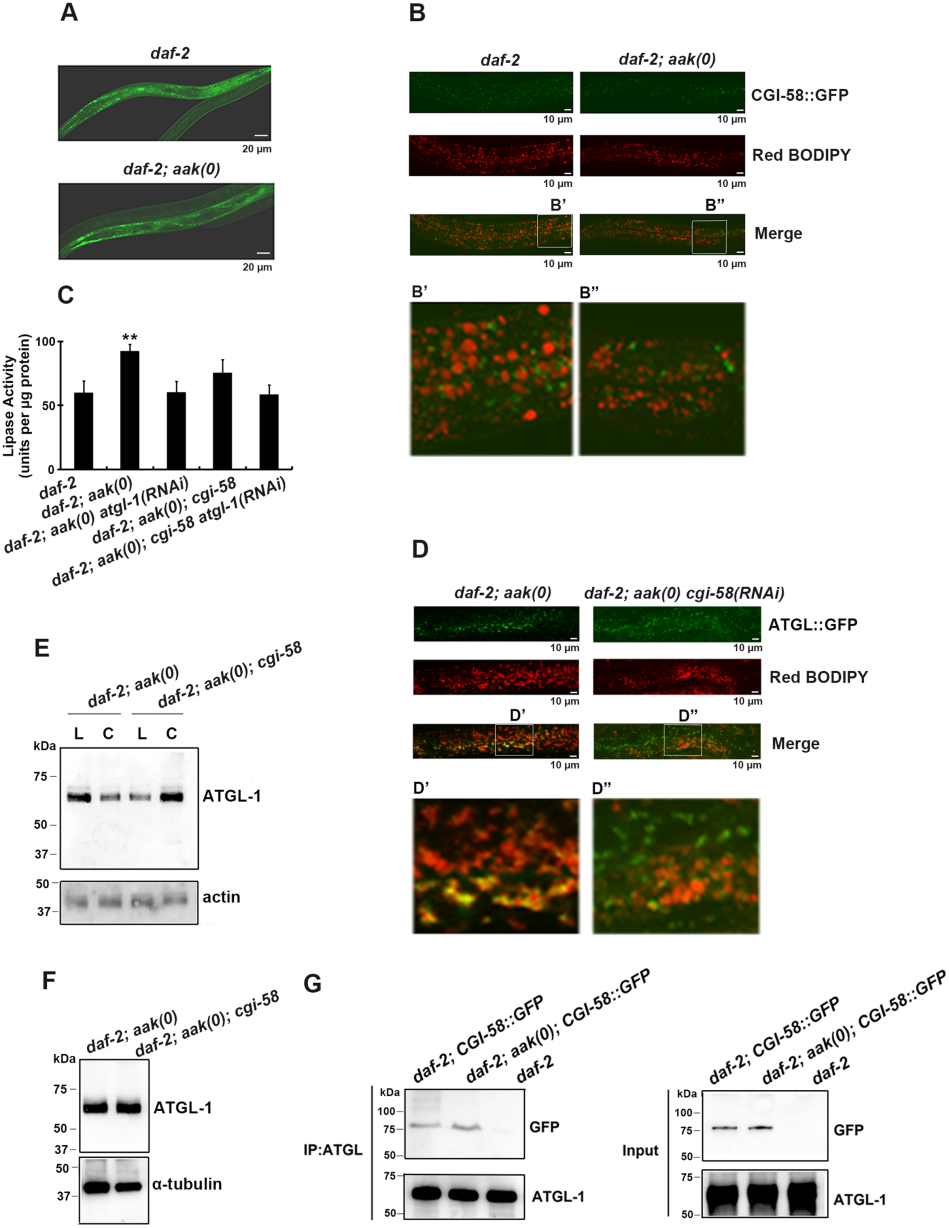
CGI-58 interacts with ATGL-1 and tethers it to the lipid droplets. **(A)** CGI-58::GFP was expressed in the hypodermis and the intestine at comparable levels in both control *daf-2* and *daf-2; aak(0)* mutant dauer larvae. Both strains carry the same *ex[Pcgi-58*::*cgi-58*::*GFP; rol-6(gf)]* transgene (See Materials and Methods). Scale bar = 20 μm. **(B)** CGI-58::GFP localizes to the surface of the lipid droplets at dauer day 0 (48 hours after being shifted to 25°C at the L1 stage) in both *daf-2* and *daf-2; aak(0)* mutant dauers. Both strains carry the same *ex[Pcgi-58*::*cgi-58*::*GFP; rol-6(gf)]* transgene. Scale bar = 10μm. Insets (B' and B") were generated by selecting the same size of frame for each image followed by proportionate amplification to the same magnification. **(C)** Optimal ATGL-1 lipase activity requires *cgi-58*. ATGL-1-dependent lipase activity was determined in *daf-2; aak(0)* mutant dauer larvae with wild type or compromised *cgi-58/atgl-1* function. ** indicates statistical significance (P<0.01) compared to all three of the other genotypes. Error bars indicate SD of three independent experiments. **(D)** Elimination of *cgi-58* resulted in the dissociation of ATGL-1 from the lipid droplets. Both strains carry the same *hjIs67[Patgl-1*::*atgl-1*::*GFP]* transgene. Scale bar = 10μm. Insets (D' and D") were generated by selecting the same size of frame for each image followed by proportionate amplification to the same final magnification. **(E)** ATGL-1 association with the lipid droplets is dependent on appropriate CGI-58 levels. Immunoblot analysis was used to determine the levels of ATGL-1 in isolated lipid droplets (L) and cytoplasm (C) obtained from total day 0 dauer extracts of each genotype. Protein concentration was measured and 30μg of total protein was loaded in each sample lane. Actin was used as a loading control for the total protein level. **(F)** CGI-58 does not contribute to ATGL-1 stability in AMPK mutant dauers. ATGL-1 levels were determined by immunobloting using anti-ATGL-1 antisera in lysates obtained from AMPK mutants with or without *cgi-58*. **(G)** CGI-58 physically interacts with ATGL-1 in vivo in both control and AMPK mutant dauers. Co-immunoprecipitations were performed with *daf-2* and *daf-2; aak(0)* day 0 dauer larvae carrying the same *ex[Pcgi-58*::*cgi-58*::*GFP;rol-6(gf)]* transgene using anti-ATGL-1 serum for pull down and blotted with anti-GFP serum.

To better understand the role of *C*. *elegans* CGI-58 in regulating ATGL-1, we generated a CGI-58::GFP translational fusion transgene and introduced it into control *daf-2* animals (*daf-2* dauer larvae will hitherto be referred to as control dauers) that were AMPK-deficient. During the dauer stage, *C*. *elegans* CGI-58 is expressed in both control and AMPK mutant dauer larvae mainly in the hypodermis and intestine (Fig 2A), the two tissues that have been well characterized for their role in lipid synthesis and storage. Next, we stained the lipid droplets of these animals with red C_1_-BODIPY-C_12_ and found that most of the GFP-tagged *C*. *elegans* CGI-58 proteins are closely associated with the lipid droplets in both control *daf-2* and AMPK-deficient dauer larvae (B' and B" in Fig 2B).

Given that loss of *C*. *elegans* CGI-58 increased the triglyceride level in AMPK-deficient dauer larvae and the well-characterized role of it as a co-activator of ATGL in mammals, we next questioned whether *C*. *elegans* CGI-58 affects the lipase activity of these animals. Using a colorimetric assay to measure lipase activity, we found that the lipase activity was significantly reduced in the absence of *C*. *elegans* CGI-58, which is solely attributed to ATGL-1, since no additional effect was observed when we eliminated both *cgi-58* and *atgl-1* compared to the loss of *atgl-1* alone (Fig 2C).

To determine whether the reduced lipase activity is due to failure of the ATGL-1 proteins to attach to the lipid droplets in the absence of CGI-58 we used a transgenic strain that expressed a fully functional ATGL-1::GFP translational fusion protein in a *daf-2; aak(0)* background. We noted that the ATGL-1::GFP signal was closely associated with the red C_1_-BODIPY-C_12_-stained lipid droplets, while the signal was distinct from the lipid droplets in the mutant dauer larvae that lacked *cgi-58* (D' and D" in Fig 2D). To further confirm the role of *C*. *elegans* CGI-58 in localizing ATGL-1 to the lipid droplets, we isolated lipid droplets from intact animals and determined the ATGL-1 protein levels in these AMPK-deficient dauer larvae that were competent or compromised for CGI-58 function. The isolated lipid droplet samples were verified by triglyceride analysis and C_1_-BODIPY-C_12_ staining (see Materials and Methods), (S2 Fig) and following separation we observed that ATGL-1 was more abundant in the lipid droplet fraction compared to our cytoplasmic fractions prepared from AMPK-deficient dauer larvae. However, this was completely reversed in the isolated fractions from otherwise genotypically-identical animals that lacked *C*. *elegans* CGI-58 (Fig 2E), suggesting that ATGL-1 associates with the lipid droplets in AMPK-deficient dauer larvae in a CGI-58-dependent manner.

To determine whether *C*. *elegans* CGI-58 might contribute to the stability of ATGL-1 in this developmental context we compared ATGL-1 levels in AMPK-deficient and AMPK; CGI-58-deficient dauer larvae using an antibody that we generated against ATGL-1 (S3 Fig), and noted that overall ATGL-1 protein levels were unaffected by the elimination of CGI-58 (Fig 2F).

In mammalian cells CGI-58 was reported to bind to ATGL, which leads to its optimal activation in vitro [20]. We therefore tested whether *C*. *elegans* CGI-58 interacts with ATGL-1 in vivo during the dauer stage, and if so, whether this association might be regulated by AMPK. We used control *daf-2* and AMPK-deficient dauer animals that expressed a GFP-tagged *C*. *elegans* CGI-58 transgene and immunoprecipitated ATGL-1 using our antibody. By probing for CGI-58::GFP protein we noted that the interaction between *C*. *elegans* CGI-58 and ATGL-1 is not affected by the presence or absence of AMPK (Fig 2G). Taken together, *C*. *elegans* CGI-58 protein is mainly expressed in the hypodermis and intestine where it interacts with ATGL-1 to tether it to the lipid droplets in close proximity to its triglyceride substrates.

### CGI-58 Regulates Lipid Droplet Size

Given that *C*. *elegans* CGI-58 is in constant close association with lipid droplets, we wondered whether it was solely required for its well-documented enzymatic function, or whether it might impinge upon additional aspects of lipid droplet integrity when lipolysis is not active. It was recently shown that in late stage wild type larvae C_1_-BODIPY-C_12_ labels lysosome-related organelles (LROs) with high intensity, while lipid droplets stained with low intensity [21]. Although this may be true in L4 stage larvae, C_1_-BODIPY-C_12_ almost exclusively labels lipid-related sub-compartments and predominantly the lipid droplets in dauer larvae (S4 Fig).

**Fig 3:**
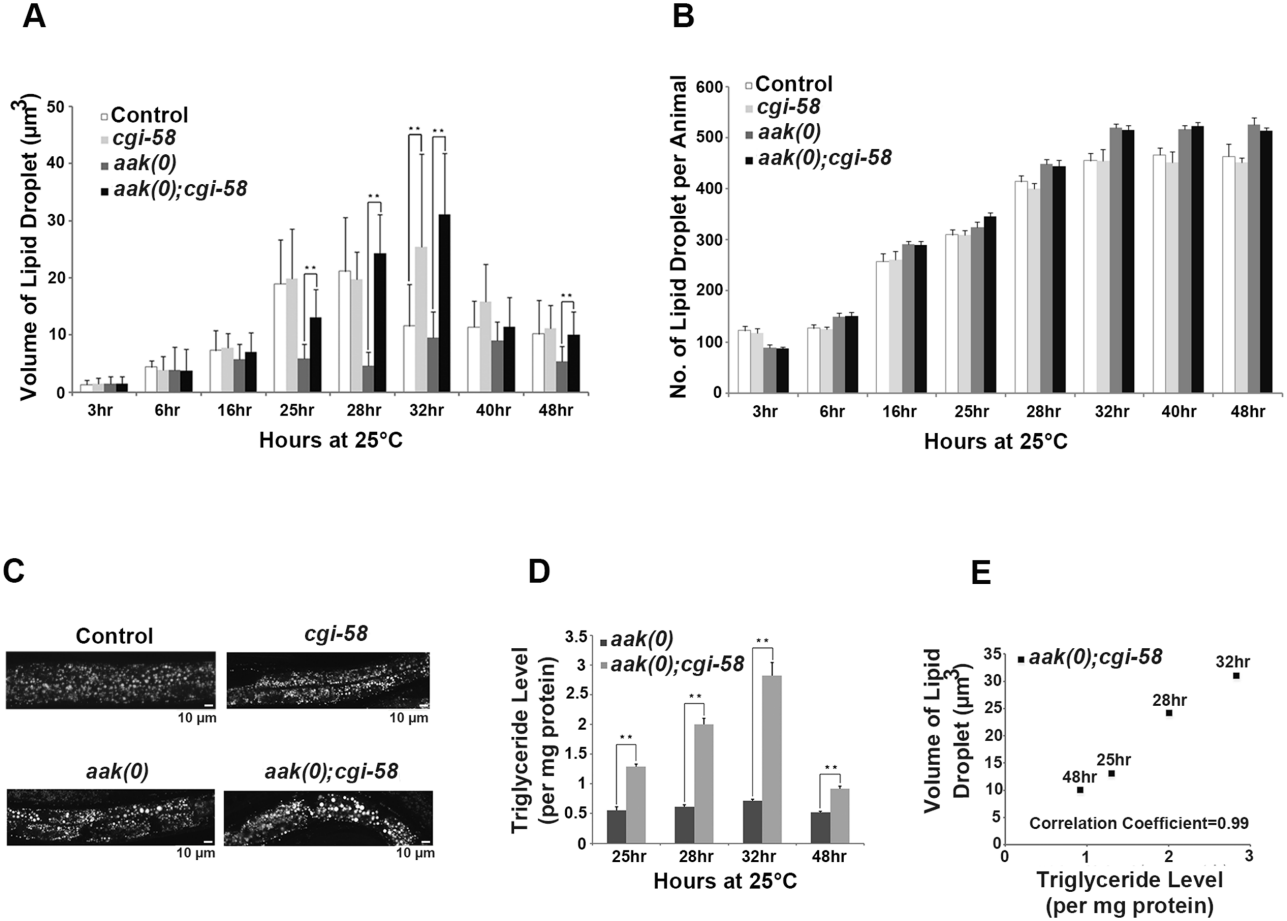
CGI-58 is essential for lipid droplet size regulation. **(A)** Elimination of *cgi-58* in *daf-2* and *daf-2; aak(0)* animals led to a dramatic increase in lipid droplet size during the later part of dauer entry. C_1_-BODIPY-C_12_ -stained lipid droplets were imaged and their dimensions were quantified using the AxioVision (Zeiss) software. ** indicates statistical significance (P<0.01). Error bars indicate SD of three independent experiments. All strains carry *daf-2(e1370)* in **(A)** to **(E)**. **(B)** Elimination of *cgi-58* in *daf-2* and *daf-2; aak(0)* animals had no effect on lipid droplet number. Error bars indicate SD of three independent experiments. **(C)** Loss of CGI-58 function caused an increase in lipid droplet size. Animals were imaged using C_1_-BODIPY-C_12_ to stain lipid droplets 32 hours after shifting to restrictive temperature (25°C) to induce dauer formation. Lipid droplet analyses performed hereafter are all at the 32 hour time point. Scale bar = 10 μm. **(D)-(E)** Increased lipid droplet size correlates with triglyceride content in dauer larvae that lack *cgi-58*. Triglyceride levels were determined in *daf-2; aak(0)* and *daf-2; aak(0); cgi-58* animals at the later part of dauer entry. ** indicates statistical significance (P<0.01). Error bars indicate SD of three independent experiments.

We therefore monitored the BODIPY-stained lipid droplets in dauer larvae to ascertain whether *C*. *elegans* CGI-58 might affect lipid droplet structure. We first documented the morphological changes that occur to the lipid droplets during the entire dauer entry period in control and AMPK-deficient animals, with or without *C*. *elegans* CGI-58 function (S5 Fig). The lipid droplet size of the CGI-58-deficient AMPK mutant animals was compared to those present in AMPK-deficient animals from 25 hours into the dauer entry period and thereafter to reveal that the size of the droplets was significantly increased in the animals that lacked *C*. *elegans* CGI-58 (Fig 3A). The difference was most pronounced 32 hours following dauer entry and was not unique to AMPK-deficient larvae since similar defects were observed between control *daf-2* dauer larvae and CGI-58-deficient dauers (Fig 3A -32hr time point and 3C).

We further confirmed these results by performing fixation-based C_1_-BODIPY-C_12_ (S6A and S6B Fig) and Oil Red O staining (S6C Fig). In addition, we compared the total number of lipid droplets of each animal during the entire dauer entry period and observed no obvious changes between CGI-58-competent and -deficient animals in both control *daf-2* and AMPK mutant backgrounds (Fig 3B), indicating that *C*. *elegans* CGI-58 is only involved in mediating the size/morphology, but not the abundance of lipid droplets.

The dauer entry period is critical for the larvae to establish their lipid stockpile and as such we documented the lipid droplet size and abundance during the early dauer stage in these animals (S7 Fig). No effect on either of these parameters was detected in control *daf-2* dauer larvae that lacked CGI-58 during the entire early dauer stage. Furthermore, we did not observe any change in these lipid droplet characteristics in AMPK mutant dauers during the first two days. Therefore we believe that CGI-58 function is critical to regulate lipid droplet size during the dauer entry period when lipid synthesis is active. A sharp drop in both the size and number of lipid droplets was observed in day 3 and 4 AMPK mutant dauers likely due to the misregulated ATGL-1 activity that occurs in the absence of AMPK. Taken together, our data suggest that CGI-58 regulates lipid droplet size and morphology predominantly during the dauer entry period when the larva is accumulating its triglyceride stockpile.

Since we observed a persistent increase in lipid droplet size in AMPK mutant dauer larvae that lacked *C*. *elegans* CGI-58 we questioned whether the increase resulted from an accumulation of triglycerides simply due to reduced hydrolytic activity. We measured the triglyceride level in isolated lipid droplets from these animals at the corresponding time points and found that the abundance of triglycerides correlated with the increase in lipid droplet size at all time points that were analysed (Fig 3D and 3E).

The increased lipid droplet size observed in AMPK; CGI-58-deficient animals is most probably due to reduced ATGL-1 activity rather than blockage of ER flux since the lipid droplets of all the genotypes we tested that affected ER flux showed an initial gradual increase in size indicating a constant deposit of synthesized lipids until about 32 hours into the dauer entry period, after which the size began to decrease, probably marking the termination of feeding and corresponding lipid synthesis (Fig 3A and S5 Fig). If ER flux were affected, the initial increase in lipid droplet size would likely not occur due to the impaired transport machinery. Therefore, taken together, our results suggest that the presence of the ATGL-1/CGI-58 complex is crucial for the maintenance of the normal spherical morphology and volume of the lipid droplets, which is dependent on triglyceride deposition following synthesis in the ER.

### CGI-58 Prevents Lipid Droplet Fusion in An ATGL-1-Independent Manner

**Fig 4:**
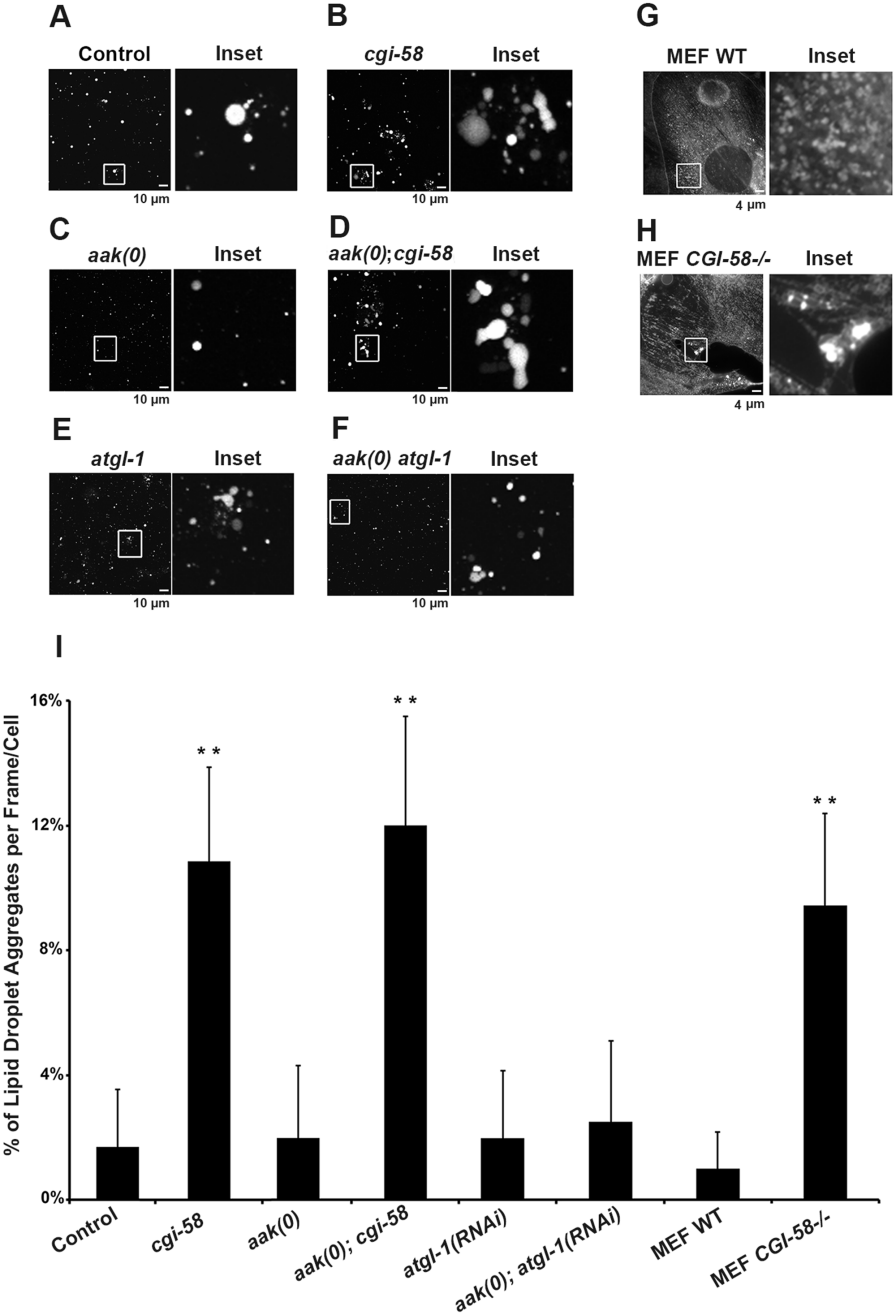
Increased lipid droplet aggregates were observed in CGI-58-deficient Dauers. **(A)-(F)** *cgi-58*, but not *atgl-1*, limits the formation of lipid droplet aggregates in both control and AMPK mutant dauer larvae. Dauer larvae were stained with C_1_-BODIPY-C_12_ 32 hours after being shifted to 25°C. Lipid droplets were subsequently isolated from the stained dauer larvae. Scale bar = 10 μm. All strains carry *daf-2(e1370)* in **(A)-(F) and (I)**. **(G)-(H)** *CGI-58* limits the formation of lipid droplet aggregates in MEFs. MEFs were stained with C_1_-BODIPY-C_12_ and imaged using a 100x objective. Scale bar = 4 μm. **(I)** Quantification of the percentage of lipid droplet aggregates per frame (for dauer larvae) or per cell (for MEFs) described in **(A)-(H)** determined for 1000 lipid droplets/100 cells analyzed for each respective genotype. Lipid droplets with a volume greater than 20 μm^3^ are considered as aggregates. ** indicates statistical significance (P<0.01) to all other columns.

Despite continuous ER-mediated triglyceride influx wild type lipid droplets maintain a homogenous size and shape that reflects the physiological state of the cell [22]. Blocking lipolysis by compromising ATGL-1 activity could therefore be exclusively responsible for the observed increase in droplet size. However, while monitoring the changes in lipid droplet morphology we noticed that the loss of *C*. *elegans* CGI-58 led to an increased frequency of lipid droplet encounters in isolated lipid droplets of both control *daf-2* and AMPK-deficient animals (Fig 4A, 4B, 4C, 4D and 4I). Interestingly, this increase in encounter frequency was never observed in ATGL-1-deficient control or AMPK mutant animals (Fig 4E, 4F and 4I), indicating that *C*. *elegans* CGI-58 possesses some ATGL-1-independent function that restricts lipid droplet association in order to maintain droplet size and shape.

**Fig 5:**
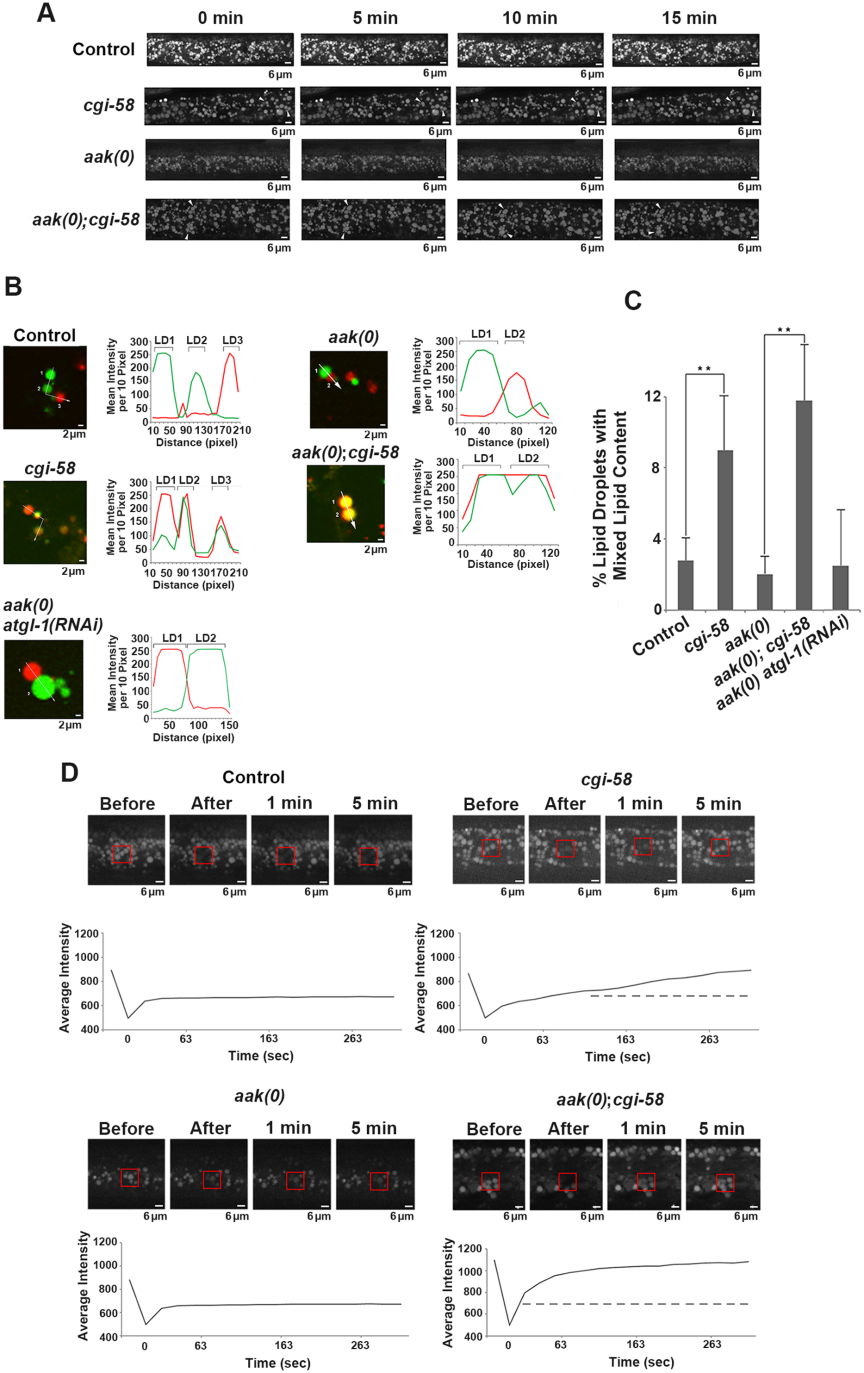
CGI-58 regulates lipid droplet fusion. **(A)** *cgi-58* restricts fusion events among lipid droplets in both control and AMPK mutant dauer larvae. Red C_1_-BODIPY-C_12_ labeled lipid droplets were imaged in real time (15 min) in control *daf-2*, *daf-2; cgi-58*, *daf-2; aak(0)* or *daf-2; aak(0); cgi-58* mutant dauer larvae 32 hours after shifting to restrictive temperature. Scale bar = 6 μm. All strains carry *daf-2(e1370)* in **(A)-(D). (B)** *cgi-58*, but not *atgl-1* limits the exchange of lipid content among lipid droplets isolated from control, CGI-58, AMPK and AMPK; CGI-58 mutant dauer larvae 32 hours after shifting to restrictive temperature. Lipid exchange was determined between isolated Green and Red C_1_-BODIPY-C_12_ labeled lipid droplets mixed in vitro for 30 minutes, following which fluorescence of individual droplets was analysed. Longer incubation times were performed and similar results were obtained. The abundance of lipid droplets/ml reaction volume was adjusted to similar levels prior to quantification. LD1 and LD2 represent two individual differentially labelled lipid droplets. The line graph at the right side of each set of images represents the fluorescent intensity of Green and Red C_1_-BODIPY-C_12_ labeled lipid droplets along the white arrow. Scale bar = 2 μm. **(C)** Quantification of the lipid droplets containing both green and red labelled lipid contents (both red and green fluorescent intensities are greater than 100) derived from the droplets described in **(B)** determined for 1000 lipid droplets analyzed for each respective genotype. ** indicates statistical significance (P<0.01). **(D)** Lipid content was more rapidly replenished in droplets obtained from animals that lack CGI-58. The dotted line in the graphs of the second (*cgi-58*) and fourth (*aak(0); cgi-58*) panel represent the maximum recovered intensity observed in the graph of the first (control *daf-2*) and third (*aak(0)*) panel. FRAP analysis was performed on control *daf-2*, *daf-2; cgi-58*, *daf-2; aak(0)* or *daf-2; aak(0)*; *cgi-58* dauer day 0 animals. Raw imaging data were captured and processed using Metamorph image acquisition software. Scale bar = 4 μm.

It is possible that the increased frequency of lipid droplet encounters might only occur in isolated droplets and have little relevance to droplet behavior in vivo. To address this we monitored lipid droplet dynamics in live dauer larvae in real time. By imaging live animals over a period of 15 min we observed an enhanced frequency of encounters that occurred among individual lipid droplets in CGI-58-deficient control *daf-2* and AMPK mutant animals (Fig 5A and S1–S4 Movies).

CGI-58 was identified as the causative gene in the rare lipid storage disease Chanarin-Dorfman Syndrome [12], but despite its well-documented role as a cofactor for optimal ATGL-mediated lipolysis, the phenotype of the CGI-58-/- mutant mouse is not identical to the ATGL-/- mouse, suggesting that it fulfills other cellular functions that are likely independent of ATGL in mammals. Since we revealed an ATGL-1-independent function of *C*. *elegans* CGI-58 in regulating lipid droplet association in *C*. *elegans* we questioned whether this unique function of *C*. *elegans* CGI-58 might be conserved in higher animals. We therefore examined lipid droplets in CGI-58-deficient mouse embryonic fibroblasts (MEFs) and noted that the droplets in the mutant MEFs also showed a similar increased frequency of lipid droplet encounters (Fig 4G, 4H and 4I), suggesting that CGI-58 may play an evolutionarily conserved role in restricting lipid droplet encounters and potentially coalescence from *C*. *elegans* to mouse.

Although we observed a significant increase in the frequency of lipid droplet encounters in the CGI-58 compromised animals, we could not address whether these events were associated with an exchange of lipid contents. To better understand the consequences of these lipid droplet encounters and how CGI-58 affected them, we differentially labeled the lipid droplets of two populations of animals with either red or green BODIPY, respectively. We then isolated the two sets of lipid droplets, co-incubated them, and documented the exchange of differentially-labelled lipid constituents among the lipid droplets isolated from control or AMPK-deficient dauer larvae with and without functional *C*. *elegans* CGI-58. We noticed that loss of *C*. *elegans* CGI-58, but not ATGL-1, resulted in an enhanced exchange of lipid content among the droplets in both control and AMPK-deficient animals (Fig 5B and 5C). Representative lipid droplets containing both red and green BODIPY-labeled lipids are highlighted with arrowheads and the fluorescent intensity of each channel was quantified to illustrate the lipid exchange event during the 30min co-incubation excluding the possibility of fluorescence overlap. To further evaluate whether a measurable exchange of lipid was indeed occurring during these encounters we employed FRAP analysis to quantify the turnover rate of fluorescently labelled lipid constituents in the droplets isolated from our various genetic backgrounds. If fusion events between the droplets were occurring and lipid was being exchanged, then a photo-bleached fluorescently-labelled region of the droplet should be replenished with non-bleached fluorescent lipid at a higher rate than if no exchange were to occur. When we examined fluorescence recovery after photobleaching regions of the lipid droplets in *C*. *elegans* CGI-58-compromised and competent animals we noted that the fluorescence recovered significantly less efficiently *in vivo* when either control *daf-2* or AMPK-deficient dauer larvae possessed functional *C*. *elegans* CGI-58 indicating that *C*. *elegans* CGI-58 not only restricts the frequency of lipid droplet encounters but also ensures that exchanges of lipid constituents are restricted between droplets (Fig 5D).

### CGI-58 Prevents Lipid Droplet Fusion by Altering Fatty Acid Composition and Abundance in the Phospholipid and Triglyceride Partitions of the Lipid Droplets

**Fig 6:**
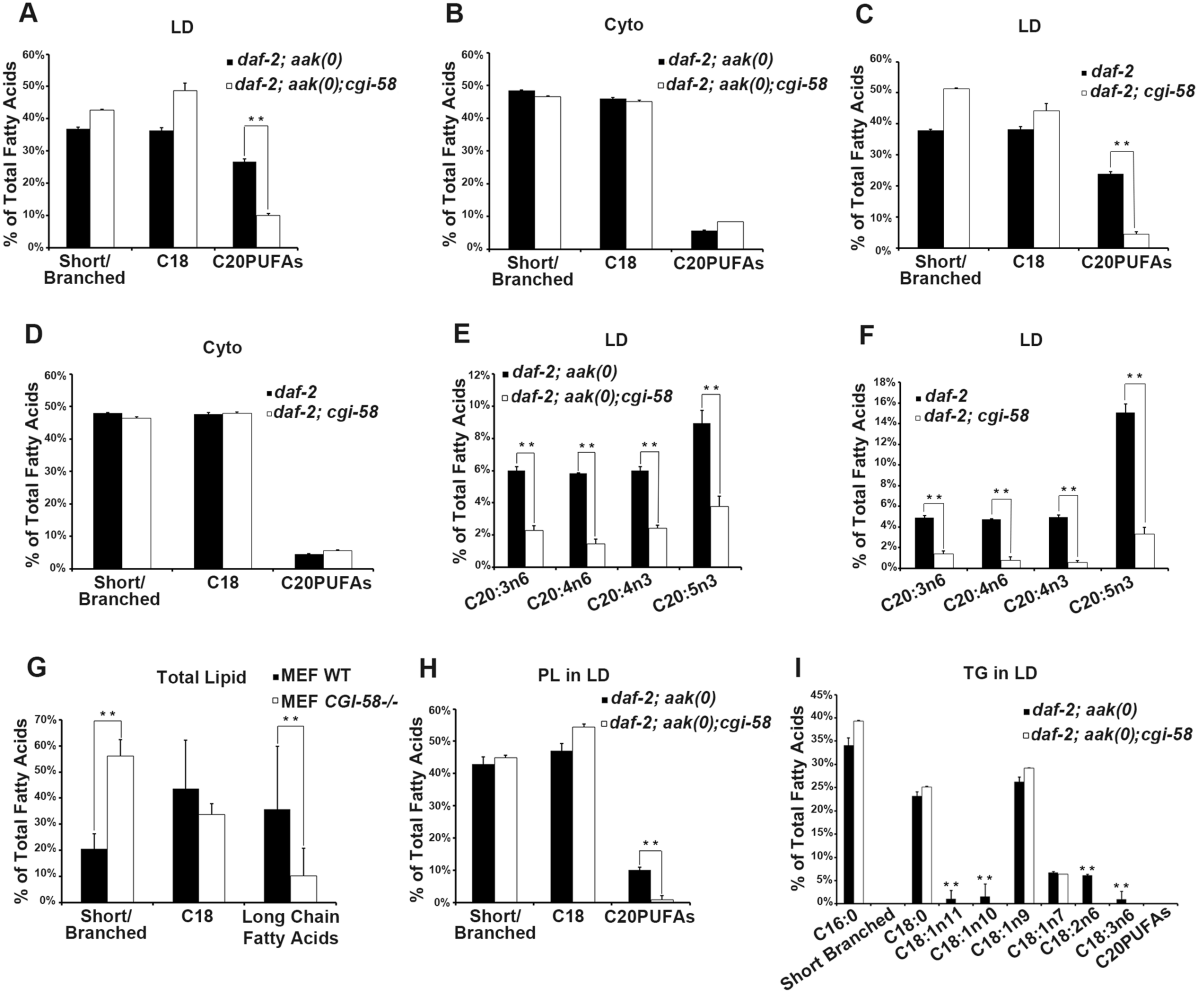
CGI-58 affect C20PUFA abundance in the lipid droplet membrane. **(A)-(D)** Loss of *cgi-58* in AMPK mutant (**A** and **B**) and control *daf-2* (**C** and **D**) dauer larvae led to a lipid droplet-specific reduction of C20PUFAs. LD = lipid droplets. Cyto = cytoplasm. Purified lipid droplets and remaining cytoplasm were subjected to fatty acid methyl ester (FAME) extraction followed by GC/MS analysis. Short/branched fatty acids are the combined total percentage of C14:0, C15ISO, C16ISO, C16:0, C17ISO, C16:1n7 and C17Δ. C18 fatty acids are the combined total percentage of C18:0, C18:1n11, C18:1n10, C18:1n9, C18:1n7, C18:2n6 and C18:3n6. C20PUFAs are total percentage of C20:3n6, C20:4n6, C20:4n3 and C20:5n3. ** indicates statistical significance (P<0.01) and error bars indicate SD of three independent experiments in **(A)-(I)**. **(E)-(F)** The amount of all major C20PUFAs were decreased in the lipid droplets of *daf-2; aak(0); cgi-58* **(E)** and *daf-2; cgi-58* **(F)** dauer larvae. **(G)** Long chain fatty acids were decreased in *CGI-58* MEF mutants. Total cell lysates were subjected to FAME extraction followed by GC/MS analysis. Long chain fatty acids include fatty acids with a carbon chain longer than 20 and certain aromatic fatty acids. **(H)** C20PUFAs were lost from the phospholipid portion of the lipid droplets of *daf-2; aak(0); cgi-58* dauer larvae. PL = phospholipid. **(I)** Some C18 fatty acids were completely absent in the triglyceride portion of the lipid droplets of *daf-2; aak(0); cgi-58* dauer larvae. TG = triglyceride.

It is not clear how *C*. *elegans* CGI-58 might affect the fusion of lipid droplets *in vivo* based on our current understanding of its function. Several parameters could affect the rate of lipid exchange including membrane fluidity and/or lipid composition. To further understand how *C*. *elegans* CGI-58 prevents lipid droplet fusion we determined the lipid composition of isolated lipid droplets from AMPK mutant dauer larvae that were either compromised or competent for *C*. *elegans* CGI-58 function using GC/MS analysis. We found that in the absence of *C*. *elegans* CGI-58, the relative amount of total C20PUFAs was significantly reduced in the lipid droplets of both AMPK mutant (Fig 6A) and control *daf-2* dauers (Fig 6C), due to the general decrease in abundance of all the individual species of *C*. *elegans* C20PUFA of varying degrees of saturation (Fig 6E and 6F). Moreover, this reduction was only observed in isolated lipid droplets, but not in the total lysates, suggesting a lipid droplet-specific role of *C*. *elegans* CGI-58 in this context (Fig 6B and 6D).

To test whether mammalian CGI-58 might affect lipid composition in the droplets in a comparable manner we repeated our analysis with lipid extracts from *CGI-58*-compromised MEFs. Interestingly, we observed a similar reduction in the amount of long chain fatty acids including fatty acids with carbon chains >C20 in addition to certain aromatic fatty acids (Fig 6G), indicating that the conserved role of CGI-58 in regulating lipid droplet fusion may be mediated through effects on fatty acid composition within the droplets *per se* both in *C*. *elegans* and mice.

To determine whether the loss of *C*. *elegans* CGI-58 and the consequent change in fatty acid composition affected the neutral lipid core or the polar lipid constituents within the droplet membrane we purified phospholipids (monolayer membrane) and triglyceride (core content) from isolated lipid droplets and analysed and compared the composition of each compartment of the organelle. GC/MS analysis revealed that the loss of C20PUFAs occurred exclusively within the phospholipid compartment (Fig 6H), where these long chain fatty acids are necessary components to maintain fluidity within the membrane environment [23]. In addition, we also noted that a significant number of C18 fatty acids, many of which are necessary precursors of C20PUFAs, were completely absent in the triglyceride compartment of CGI-58-deficient AMPK mutant animals (Fig 6I), which might explain the loss of C20PUFAs in the phospholipid compartment. Conversely, the overall abundance of individual C18 fatty acid species remained unchanged in the phospholipid compartment of these animals. We also observed a complete absence of C20PUFAs in the triglyceride compartment of both CGI-58-competent and-deficient animals, possibly due to their contribution to the phospholipid components that comprise and ultimately determine the physical properties of the membrane.

**Fig 7:**
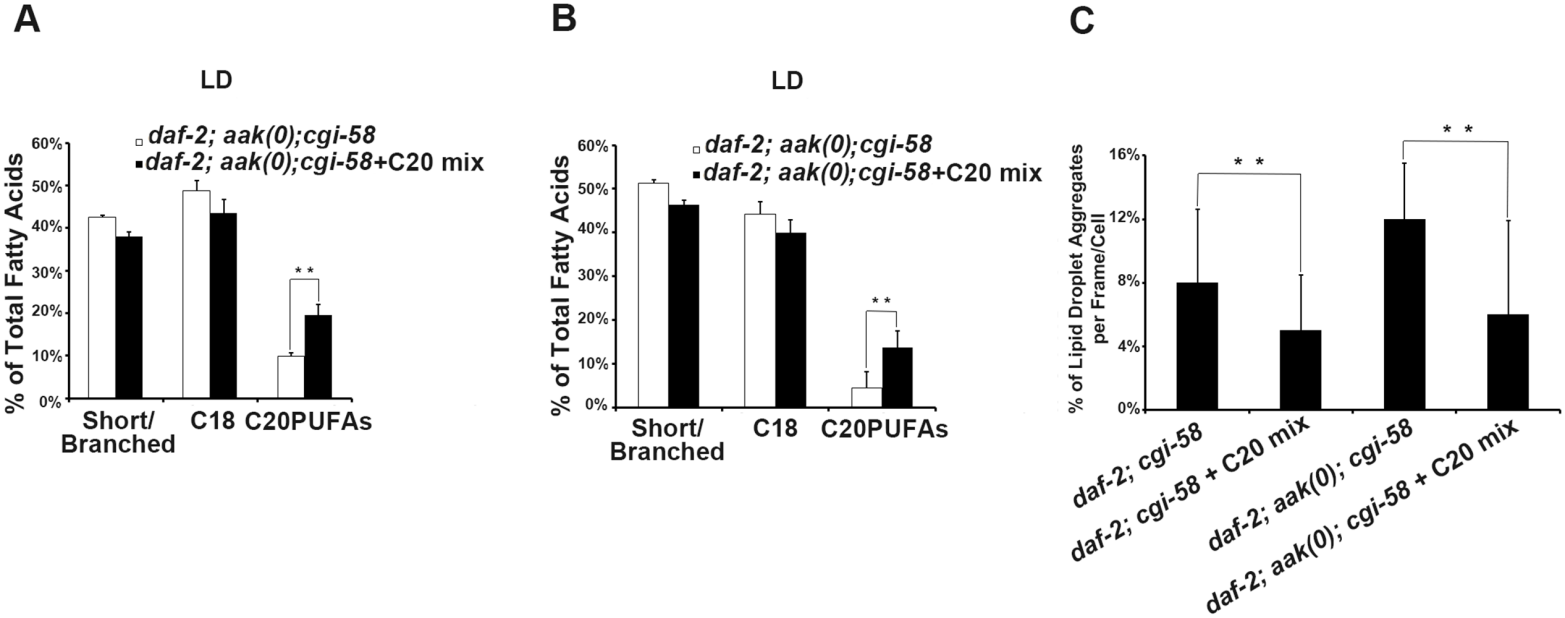
Dietary C20PUFA supplementation reduced the number of lipid droplet aggregates in CGI-58 and AMPK; CGI-58 mutants. **(A)-(B)** Dietary supplementation of C20PUFA enriched the lipid droplet C20PUFA composition of *daf-2; aak(0); cgi-58* **(A)** and *daf-2; cgi-58* **(B)** dauer larvae represented by their increased C20PUFA portion. **(C)** Less lipid droplet aggregates were observed in both the *daf-2; cgi-58* and *daf-2; aak(0); cgi-58* animals following C20PUFA dietary supplementation. Lipid droplets with a volume greater than 20 μm^3^ are considered as aggregates.

If indeed the reduced C20PUFA composition of the lipid droplet contributes to the coalescence behaviours that we observe in the AMPK mutant animals that lack CGI-58 then increasing their concentration should improve the defect in the mutant lipid droplets. We therefore supplemented the food with C20PUFAs and fed this mixture to both animals that lacked CGI-58 and those that were mutant for both AMPK and CGI-58. The supplementation successfully enriched the C20PUFA concentration in the lipid droplets isolated from both groups (Fig 7A and 7B), and which led to a reduced number of lipid droplet aggregates in these animals (Fig 7C). These results suggest that in *C*. *elegans* CGI-58 is essential to maintain the abundance of certain fatty acid species in both the triglyceride (C18s) and phospholipid (C20PUFAs) compartments, where the former serve as precursors for the latter. Because the composition of long chain fatty acids present on the lipid droplet surface can affect numerous physical parameters of the organelle including the intrinsic curvature of connected lipid droplets that determines further expansion or closure of the connected pores [24], it is not inconceivable that the effects of *C*. *elegans* CGI-58 impinge on lipid composition of the droplet monolayer, which indirectly blocks droplet fusion in AMPK mutant dauer larvae.

### Variations in Phosphatidic Acid Levels Reduced C20PUFAs Production

Previous studies in yeast have shown that CGI-58 possesses lysophosphatidic acid acyltransferase (LPAAT) activity that catalyzes the formation of phosphatidic acid (PA) [25], while the LPAAT activity of CGI-58 is currently contentious in mammals [26], [27].

**Fig 8:**
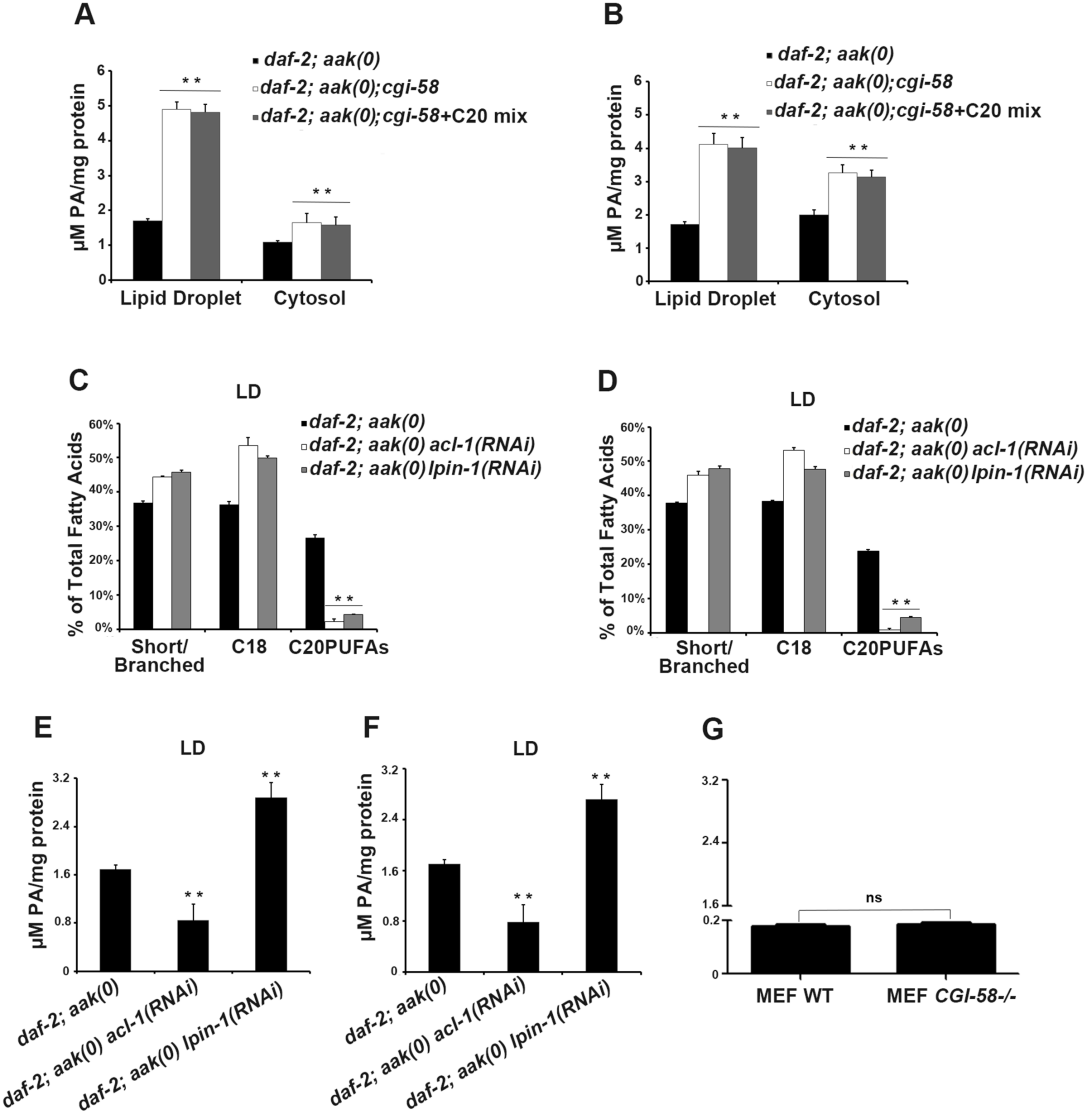
Variation in phosphatidic acid (PA) levels is associated with reduced C20PUFA abundance. **(A)-(B)** PA levels are low in both the lipid droplets and cytoplasm of *daf-2; aak(0); cgi-58* **(A)** and *daf-2; cgi-58* **(B)** dauer larvae, and are not affected by dietary supplement of C20PUFAs. **(C)-(F)** Fluctuation in PA levels achieved by eliminating enzymes involved in either PA turnover (LPIN-1), or biosynthesis (ACL-1) resulted in reduced C20PUFA levels in the lipid droplets of *daf-2; aak(0)* (C), (E) and *daf-2* (D), (F) dauer larvae. LD = lipid droplet. **(G)** PA levels were unchanged in *CGI-58* MEF mutants.

Given that PA serves as a signaling lipid that recruits a number of cytosolic proteins to the membrane [28], [29], while also acting as a negatively curved lipid that tends to stabilize the pore between connected lipid droplets to favor pore formation and/or maintenance [24], we questioned whether loss of *C*. *elegans* CGI-58 might also alter the PA level in lipid droplets. Indeed, we found that loss of *C*. *elegans* CGI-58 in both AMPK mutant (Fig 8A) and control *daf-2* (Fig 8B) dauer larvae led to increased PA levels in both lipid droplets and in the cytoplasm.

It is not clear whether the increased PA level is a causative factor or the result of the reduced C20PUFA abundance in the *C*. *elegans* CGI-58 compromised lipid droplets so we eliminated LPIN-1, the *C*. *elegans* orthologue of the mammalian PA phosphohydrolase; a critical enzyme required for PA degradation that catalyzes the conversion of PA to diacylglycerol [30]. We performed *lpin-1(RNAi)* in AMPK mutant and control *daf-2* dauer larvae and subjected their lipid droplets to GC/MS analysis. We observed a similar reduction in C20PUFA abundance that was comparable to that observed in the *aak(0); cgi-58* and *cgi-58* mutants (Fig 8C and 8D), indicating that the increased PA level is likely responsible for the decrease in C20PUFA abundance. Surprisingly, we observed a similar reduction in C20PUFA levels when we eliminated ACL-1; the *C*. *elegans* orthologue of a key mammalian LPAAT enzyme that catalyzes the production of PA from lysophosphatidic acid (LPA) [31] (Fig 8C and 8D), indicating that any variation in the droplet PA homeostasis compromises the production of C20PUFAs. Moreover, dietary enrichment of C20PUFA did not affect the levels of PA present in lipid droplets of CGI-58-compromised dauers, indicating that the variation in PA concentration impacts the production of C20PUFAs by an uncharacterised mechanism and not the converse (Fig 8A and 8B).

If the variation in PA abundance affects PUFA production in mammalian lipid droplets then a similar correlation should be apparent in the WT and *CGI-58* mutant MEFs. However, despite the similar observed reduction in long chain fatty acid abundance in *CGI-58* mutant MEFs (Fig 6G), no change in PA levels was detected in *CGI-58*-compromised MEFs (Fig 8G). Given that the PA level was much lower in the MEFs compared to *C*. *elegans* dauer larvae, mammals may employ alternative mechanisms possibly involving many other intermediate molecules to regulate C20PUFA production from C18 precursors in the droplets, although it is likely that this process still requires CGI-58.

Taken together, in *C*. *elegans* CGI-58 prevents lipid droplet fusion by increasing the level of PA in the droplet membrane, which reduces the amount of C20PUFA on the droplet surface, ultimately affecting triglyceride content, droplet size, and the overall morphology of the organelle.

## Discussion

The *C*. *elegans* dauer stage provides the larva with a unique alternative to survive a multitude of environmental stresses, where instead of continuing the normal reproductive life cycle, larvae enter a motionless and non-feeding stage that is associated with global developmental arrest. This diapause-like stage illustrates how organisms have evolved specialized adaptations that permit them to readjust their metabolic state to respond to their variable environment. Astonishingly, dauer larvae have been documented to survive extremely low pH; desiccation; osmotic stress and they can survive months without external nutrient intake by augmenting fat accumulation during the dauer entry period and subsequent metabolic remodeling, including the activation of the glyoxylate cycle and increased fatty acid *de novo* synthesis [17], [32], [33].

Upon entry into dauer AMPK expression and activation are increased. This protein kinase acts as a “metabolic master switch” by sensing high intracellular AMP:ATP ratio to regulate a number of metabolic pathways that impact on behavior, stem cell quiescence [7], lifespan determination [34], [35] and lipid metabolism [16]. AMPK mutant dauer larvae die prematurely after a short period in the dauer stage, while their survival can be enhanced by removing many genes that affect lipid turnover [17].

A recent study raised the concern that *daf-2; aak-2*-deficient dauer larvae may not die prematurely as dauer larvae, but instead the dauer larvae may recover abnormally and subsequently expire from starvation [36]. In our experiments performed with null mutations of AMPK signaling we have not observed this premature recovery, most likely due to differences in genetic backgrounds or culture conditions. However, our observation that both *atgl-1(RNAi)* and *cgi-58(RNAi)* both prolong survival when analysed with our dauer trap assay are also consistent with this alternative interpretation through their capacity to preserve triglyceride reserves as an energy source during either dauer or starvation. Therefore, despite the controversy concerning the efficacy of our assay for dauer survival, the role of both *atgl-1* and *cgi-58* in regulating lipid droplet homeostasis is not in dispute. Nevertheless, in light of these observations we recognise the need to design alternative means of assessing long-term dauer survival studies using AMPK mutants.

The triglyceride stores increase substantially in AMPK-deficient dauer larvae subjected to *atgl-1*(RNAi), while the lipid droplets acquire an abnormal morphology. Lipid droplets have long been considered as inert fat particles but are now recognized as dynamic organelles that comprise a core composed of neutral lipids surrounded by a phospholipid monolayer harbouring numerous lipophilic proteins. In mammals, a number of these lipid droplet-associated proteins have been well characterized. Among them, the Perilipin family members are considered to be the most abundant proteins present on the lipid droplet surface, presumably serving to protect the stored lipids by limiting accessibility of hydrolytic enzymes during basal conditions [37]. In addition, the CIDE family proteins are also closely associated with lipid droplets and modulate fat storage and lipid droplet size in vertebrates [38]. Intriguingly, there are no clear orthologues of these protein families in *C*. *elegans*, suggesting that alternative regulatory mechanisms must exist that are independent of these well-characterized proteins, or that other proteins have been co-opted to carry out analogous functions in *C*. *elegans*.

All organisms that possess an ATGL orthologue also harbour a CGI-58-like protein that presumably functions with it. Since the *C*. *elegans* genome does not encode regulatory molecules such as Perilipin we wondered if the interaction between ATGL-1 and CGI-58 might differ in *C*. *elegans*, circumventing any requirement for the Perilipin family of proteins. By characterizing a *C*. *elegans* orthologue of the human CGI-58 protein we revealed that it affects lipid hydrolysis in an ATGL-dependent manner. Elimination of CGI-58 in AMPK-deficient dauer larvae was sufficient to prolong the survival of these animals, much like *atgl-1*(RNAi), by correcting the associated lipolytic and osmotic defects. Consistent with its role as a co-activator of ATGL, ATGL-1 lipase activity was significantly reduced in the absence of CGI-58. Moreover, in the absence of AMPK, ATGL proteins are released from sequestration and are anew capable of binding substrate. However, this binding is mediated by CGI-58 since its elimination resulted in the clear segregation of ATGL-1 from the lipid droplets in AMPK-deficient dauer larvae. Therefore, like in mammalian cells, the activation of ATGL-1 requires its interaction with lipid droplet-bound CGI-58 [15].

Using an approach based on fluorescently-labelled lipid droplet purification we revealed that in addition to its role in activating ATGL-1, CGI-58 limits the expansion of the droplet volume following droplet-droplet encounters that result in lipid exchange or fusion. Such fusion events have been previously described in mammalian cells [39], [40]. In *Arabidopsis*, the loss of the major structural lipid droplet protein Oleosin results in an increased frequency of lipid droplet fusion, very similar to the function we ascribe to CGI-58 [41]. CGI-58 appears to have adopted dual functions throughout evolution to regulate both lipid hydrolysis and the maintenance of the lipid droplet size and morphology by regulating droplet fusion. Notably, *cgi-58* does not affect lipid droplet size during non-dauer developmental stages (L1-4 and adult) when the lipid droplet size is much smaller than that observed in dauer larvae (S8 Fig), indicating a dauer-specific role of *C*. *elegans* CGI-58 in regulating lipid droplet fusion, probably due to the temporarily increased size of the organelles as the animals enter this stage.

It is somewhat puzzling that the enzymatic function of CGI-58 would be linked to a role in restricting lipid droplet expansion. However, during lipolysis various lipases are recruited directly to the surface of the lipid droplet where they interact with their substrates at the interphase of surface monolayer and the neutral lipid core. The mechanism that permits these enzymes to penetrate the lipid monolayer of the lipid droplets remains unclear, as is the export of the released free fatty acid products out of the lipid droplets. Expanded lipid droplets might accumulate fatty acid bi-products near the surface close to the site of catalysis, potentially causing product inhibition or limiting enzyme access to the triglyceride substrate core. This would select for an optimal lipid droplet size linked to enzymatic efficacy.

Intriguingly, evidence suggests that under maximum stimulation of lipolysis, the lipid droplets break down into smaller entities to increase the surface area for enzyme interaction [42]. By preventing fusion of smaller lipid droplets into larger ones, CGI-58 may indirectly enhance the efficiency of ATGL-1. CGI-58 may therefore have been co-opted during evolution to provide the structural supporting role of the Perilipin protein family in maintaining the regular spherical structure of the lipid droplets, and potentially other types of lipid-bound cellular vesicles.

Perilipin proteins belong to the lipid droplet-coating protein family, among which Perilipin 1 is the founding member and whose role is most well characterized. One major role of Perilipin 1 is to regulate lipolysis by limiting lipase access to the lipid substrates in the lipid droplet [13]. Upon β-adrenergic stimulation, Perilipin 1 is phosphorylated by protein kinase A at multiple sites [43]. The phosphorylation has a two-fold consequence: first, it induces conformational changes of Perilipin 1 that may facilitate the translocation of lipases like ATGL and HSL onto the lipid droplets; second, it liberates CGI-58 which is initially sequestered by Perilipin 1 at the lipid droplet surface. This allows CGI-58 to interact and activate the lipase activity of ATGL [44], [45]. In addition, Perilipin family proteins have also been postulated to regulate the size and expansion of lipid droplets [46]. There are no Perilipin family members identified in the *C*. *elegans* genome, so in *C*. *elegans* dauer larvae CGI-58 may adopt this Perilipin-like function as a lipid droplet coating-protein to not only tether ATGL-1 onto the lipid droplets to initiate lipolysis but also to tightly regulate the size and morphology of individual lipid droplets by preventing their coalescence.

Recent data indicate that CGI-58 does not possess LPAAT activity [27], while our data revealed a negative correlation between CGI-58 and the levels of the LPAAT product PA present in purified lipid droplets. The increased abundance of PA in CGI-58-deficient AMPK mutant animals may be due to the direct catalytic activity of CGI-58 that shifts the equilibrium to favour LPA production, or via indirect effects that affect the PA degradation pathway. Nevertheless, the resulting increase in PA abundance in CGI-58 mutants contributes to lipid droplet fusion through two potential mechanisms. First, PA is an abundant component of the lipid droplet monolayer and its intrinsic negative curvature can match the surface of a contacting lipid droplet thereby reducing the line tension and promoting fusion [24]. Therefore, increased PA levels in the lipid droplet monolayer should enhance the frequency of fusion events, consistent with our observations of droplets obtained from CGI-58-compromised mutants. Second, an optimal PA concentration in the droplet membrane may be important to recruit proteins to the droplet monolayer, including enzymes involved in fatty acid (C20PUFAs) synthesis. In this scenario, a threshold concentration may be important for signaling, while fluctuations either side of this level affects lipid homeostasis. The abundance of C18 fatty acid species in the triglyceride core of CGI-58-deficient lipid droplets was also markedly reduced, some of which are precursors of C20PUFAs, and which might account for the reduced C20PUFA levels in the monolayer. C20PUFAs are both important membrane components and precursors of signaling lipids in most animals [23], [47], [48]. The reduction of these long chain fatty acids in the droplet could in turn affect the fluidity of the monolayer and also facilitate droplet fusion. Overall, in CGI-58-deficient lipid droplets, the components of the monolayer are remodelled substantially and are composed mainly of short chain fatty acids and a significantly increased proportion of PA, conditions that would favour lipid droplet fusion.

Although we see similar changes in the C20PUFA abundance in the *CGI-58* compromised MEFs, we did not observe parallel changes in PA levels, indicating that CGI-58 compromise affects C20PUFA abundance, which in turn results in increased lipid droplet fusion events, a defect that is conserved between nematodes and mammals, although some of the intermediate players may be species-specific given that the PA level in MEFs are extremely low compared to *C*. *elegans*.

Mutation of the *cgi-58* gene is associated with the neutral lipid storage disease, CDS, characterized by the accumulation of triglycerides in the cytoplasm of multiple tissues. Curiously, the phenotype of CGI-58-/- mice has some distinct features from those described in ATGL-/- mutant mice [49], suggesting that CGI-58 possesses ATGL-independent functions. We have demonstrated a novel role of CGI-58 in the *C*. *elegans* dauer larvae where it also acts structurally to limit expansion of the lipid droplets, thereby regulating the utilization of the triglyceride energy source. The lipid droplet coalescence phenotype we describe in *CGI-58*-deficient lipid droplets and MEFs may explain the origin of the fat blocks present in tissues of CDS patients. Our work may therefore provide novel aetiological insight concerning the previously uncharacterized ATGL-independent processes that are affected in CDS patients.

## Materials and Methods

### Reagents, Strains, Plasmids and Transgenic Animals

*C*. *elegans* strains were cultured as previously described by Brenner [50]. The following alleles and strains were used: The strain RB2386 C37H5.3*(ok3245)* was obtained from CGC and subsequently crossed into CB1370 *daf-2(e1370)* and MR1000 *daf-2(e1370); aak-1(tm1944); aak-2(ok524)* strains. *ok3245* bears a 800bp deletion that occupies almost two thirds of the gene from the C-terminal, therefore, the allele is predicted to be null. C37H5.3 DNA and its upstream ~800bp (considered as its own endogenous promoter) was amplified by PCR and subsequently cloned into pPD95.77 GFP vector to generate pMR613 *(Pcgi-58*::*cgi-58*::*GFP)*. Extrachromosomal arrays of pMR613 was generated by standard microinjection into CB1370 *daf-2(e1370)* animals using *rol-6* cDNA rescue fragment as co-injection marker. Strain VS20 *hjIs67[Patgl-1*::*atgl-1*::*GFP]* (12) was obtained from CGC and subsequently crossed into CB1370 *daf-2(e1370)* and MR1000 *daf-2(e1370); aak-1(tm1944); aak-2(ok524)* strains.

Rabbit polyclonal antibody against ATGL-1 was raised using a synthetic peptide CTKRKVPDEPTTSKR (GenScript). Rabbit polyclonal antibody against GFP was raised by McGill Animal Resources Center. WT and *CGI-58-/-* mouse embryonic fibroblasts (MEFs) were gifts from Dr. Rudolf Zechner and were cultured according to their recommendation. Mouse polyclonal antibody against Actin was purchased from Abcam (ab14128).

### Feeding RNAi

Our RNAi feeding protocol was performed as previously described [51]. Briefly, synchronized L1 animals were added onto regular plates seeded with individual dsRNA-expressing bacterial clones and maintained at 15°C. Phenotypes were scored thereafter.

### Dauer Survival

Dauer survival was determined as described [7]. Dauer larvae were kept in double-distilled water. Survival was scored according to their appearance and moving response to a gentle tap on the plate.

### Oil Red O Staining of Dauer Larvae

Oil Red O staining of dauer larvae was performed as described [52]. Briefly, dauer larvae were fixed in 2% paraformaldehyde and stained with 60% Oil Red O solution. Stained dauer larvae were observed and imaged using DIC optics on a Zeiss Imager.21 microscope equipped with a Hamamatsu camera and. Optical density was determined using OpenLab software (Improvision).

### Immunoprecipitation and Western Blotting

*C*. *elegans* larvae and adults were lysed by sonication in lysis buffer (50mM Hepes pH7.5, 150mM NaCl, 10% glycerol, 1% Triton X-100, 1.5mM MgCl_2_, 1mM EDTA and protease inhibitors) and then incubated with anti-ATGL-1 antibody. Immunoprecipitation was performed with Protein-A agarose followed by immunoblotting with anti-GFP antibody. Protein concentration was determined using a NanoDrop 2000c spectrophotometer (Thermo Scientific).

### Triglyceride Quantification

Triglyceride content was determined with a commercially available kit (Sigma-Aldrich) according to manufacturer’s recommendations. The assay is based on that triglycerides are hydrolyzed by a series reactions eventually leading to production of H_2_O_2_ that reacts with 4-AAP and ESPA to produce a quinoneimine dye, whose absorbance at 540 nm is directly proportional to ttriglyceride concentration in the sample. Absorbance was measured with a NanoDrop 2000c spectrophotometer at 540 nm. All calculated triglyceride concentrations were normalized to protein concentration.

### Osmotic Resistance Assay

Osmotic resistance of dauer larvae was performed as described [16]. Briefly, survival of day 4 dauer larvae was scored after being exposed to solutions of varying NaCl concentrations for 24 hours at 25°C.

### Quantification of Lipase Activity

Lipase activity for dauer animals was measured as described [16] using a commercially available QuantiChrom kit from BioAssay Systems according to manufacturer’s recommendations. The assay is based on that SH groups formed from lipase cleavage of BALB react with DTNB to form a yellow colored product, whose intensity, measured at 412 nm, is proportional to the lipase activity in the sample. OD values were measured with a Varioskan Flash Multimode Reader version 3.00.7 at the wavelength of 412 nm.

### C1-BODIPY-C12 Staining

C1-BODIPY-C12 staining was performed as described [53]. Briefly, synchronized L1 larvae were transferred to regular plates with C1-BODIPY-C12 (Invitrogen) and grown at 25°C. MEFs were grown on cover slips coated with 0.1% gelatin. Cells were fixed in 4% paraformaldehyde for 15 min before being stained in 1μM C1-BODIPY-C12 for 20 min. Images were acquired on a LSM510 confocal microscope (Zeiss) using a x40 1.3 oil objective. Lipid droplet diameter was measured using AxioVision (Zeiss) software and volume was calculated using the following formula: 4/3 x π x (diameter/2)^3^.

### Lipid Droplet Isolation

Lipid droplet isolation was performed as described with slight modification [54]. Briefly, animals fed with OP50/OP50+C1-BODIPY-C12 were washed with 1xPBS + 0.001% Triton X-100 and subsequently collected in Buffer A (25mM Tris pH7.6, 25mM glycine, 120mM sucrose and protease inhibitors) and kept on ice for 15min. The cells were lysed by adding liquid nitrogen and grinded with a pre-chilled metal homogenizer with a tight-fit pestle for 20 strokes on ice. The homogenates were centrifuged at 1000g for 10 min at 4°C to remove cell debris. The supernatant was then collected and centrifuged at 10,0000g for 1 hour at 4°C. The top white layer containing the lipid droplets was collected, resuspended with Buffer A and centrifuged 10,0000g again for 1 hour at 4°C to avoid cytosol and other membrane compartment contamination. Isolated lipid droplets were verified by C_1_-BODIPY-C_12_ staining, protein expression pattern and triglyceride enrichment compared to the supernatant after the first spin at 10,0000g (S2 Fig) before any further analysis.

### Lipid Exchange Assay

Green and red C1-BODIPY-C12 stained lipid droplets were isolated as described above. 100μl of each of the two populations of lipid droplets were added into a 1.5ml eppendorf tube and incubated at 15°C for 30min. Lipid exchange was observed using a LSM510 confocal microscope (Zeiss) with a x40 1.3 oil objective. Incubation at 4°C and 20°C was also tested and similar results were obtained. Fluorescent intensity was quantified using ImageJ software.

### Lipid Droplet Fusion Live Imaging

C1-BODIPY-C12 stained animals were paralyzed in 3mM levamisol for 3 min before being transferred onto a freshly prepared 2% agarose pad. Imaging was performed on dauer larvae 32 hours after shifting to restrictive temperature using a Quorum WaveFX spinning disk confocal system, on a Leica DMI6000B inverted microscope equipped with a 63x/1.40–0.6 oil objective and controlled by Metamorph acquisition software. For each animal, a time series of 15 minutes was taken.

### Fluorescence Recovery after Photobleaching (FRAP)

FRAP experiments were performed on dauer day 0 animals on a Quorum WaveFX spinning disk confocal system, on a Leica DMI6000B inverted microscope using a 63x/1.40–0.6 oil objective and controlled by the Metamorph acquisition software. For each animal, a time series was taken. After the tenth frame, 1000ms of photobleaching was performed using a mosaic laser at maximum power. The time series continued for 16 seconds/50 frames, immediately followed by0 a 5 minutes time lapse, 20 seconds per frame. Average fluorescence intensity of the bleached area was plotted against the time series. At least five photobleaching assays were performed for each strain.

### Lipid Purification and Gas Chromatography/Mass Spectrometry Analysis (GC/MS)

Phospholipid and triglyceride purification was performed as described [33] using solid phase exchange column (100mg capacity, Fischer Scientific). Purified lipids were dried and subjected to fatty acid methyl ester (FAME) extraction as described [55]. GC/MS was performed with the same setting as our previous study [17].

### PA Assay

Protein concentrations of the isolated lipid droplets and cytoplasm were determined using a NanoDrop 2000c spectrophotometer. PA assay was performed using a commercially available kit (Cayman Chemical) according to manufacturer's recommendations. The assay is based on that the PAs within the sample are hydrolyzed by lipase leading to generation of H_2_O_2_ that reacts with ADHP to yield a highly fluorescent compound resorufin, whose fluorescence intensity measured using excitation wavelengths of 530–540 nm and emission wavelengths of 585–595 nm is proportional to the PA concentration. The final PA concentration (μM) was normalized to the amount of protein in the sample (mg).

### Dietary Supplementation of C20PUFAs

Dietary supplementation of C20PUFAs was performed as described [56]. Briefly, a mixture of C20PUFA FAMEs containing equal weight of C20:1, C20:2, C20:3n6 and C20:5n3 (Sigma 18912-1AMP) was dissolved in double distilled water at a stock solution of 100mM before being added into autoclaved NGM media pre-cooled to 55°C to make a final fatty acid concentration of 0.3mM. The C20PUFA containing NGM plates were allowed to solidify in the dark for two days before being seeded with OP50 bacteria and synchronized L1 animals were added onto the plates two days later and shifted to 25°C for dauer induction.

## Supporting Information

S1 Fig Elimination of *lid-1* or C37H5.2 in AMPK mutants did not extend their Dauer survival or lipid droplet size. **(A)** *lid-1* or C37H5.2 RNAi did not increase the survival of AMPK mutant dauer larvae, while *lid-1* RNAi slightly increased the survival of AMPK; CGI-58 mutant dauers (P = 0.0223). **(B)**-**(C)** *lid-1* or C37H5.2 RNAi did not affect the lipid droplet size in either AMPK or AMPK; CGI-58 mutant dauer larvae.
(TIF)

S2 Fig Lipid droplet isolation-method verification. C_1_-BODIPY-C_12_ staining of isolated lipid droplets and cytoplasm (remaining portion of the total lysate) from *daf-2* day 0 dauer larvae. Associated proteins present in the isolated lipid droplets were clearly distinct from that of the supernatant and the cellular debris fractions. Triglyceride was enriched several-fold in the isolated lipid droplets.
(TIF)

S3 Fig *C*. *elegans* specific ATGL-1 antibody verification. The Anti-ATGL-1 antibody is specific for ATGL-1. The ATGL-1 antisera recognizes a single band that migrates at approximately 70kD, which corresponds to the molecular weight of ATGL-1, and is reduced in *atgl-1(RNAi)* animals.
(TIF)

S4 Fig C_1_-BODIPY-C_12_ only stains lipid droplet in Dauer larvae. C_1_-BODIPY-C_12_ staining of lipid droplets demonstrated a similar staining pattern in *daf-2* and *daf-2; glo-4* (lack LROs) Day 0 Dauer Larvae, indicating that C_1_-BODIPY-C_12_ only stains lipid droplets in dauer larvae.
(TIF)

S5 Fig C_1_-BODIPY-C_12_ staining of lipid droplets during the entire Dauer entry period of *daf-2*, *daf-2; cgi-58*, *daf-2; aak(0)* and *daf-2; aak(0)*; *cgi-58* animals. (TIF)

S6 Fig Comparative staining of lipid droplets. *daf-2*, *daf-2; cgi-58*, *daf-2; aak(0)* and *daf-2; aak(0)*; *cgi-58* Animals at the 32 hours Dauer Entry Time Point were stained either using C_1_-BODIPY-C_12_ [(A) or (B)] Oil Red O (C). No differences between the methods were observed.
(TIF)

S7 Fig C_1_-BODIPY-C_12_ staining of lipid droplets during the early Dauer stage (Day 1 to 4) of *daf-2*, *daf-2; cgi-58*, *daf-2; aak(0)* and *daf-2; aak(0)*; *cgi-58* animals (A), quantification of lipid droplet volume (B) and number (C). (TIF)

S8 Fig C_1_-BODIPY-C_12_ staining of lipid droplets at all developmental stages of N2 and *cgi-58(ok3245)* animals (A) *daf*, *daf-2; cgi-58*, *daf-2; aak(0)* and d*af-2; aak(0); cgi-58* animals (C) and quantification of lipid droplet volume (B) and (D). (TIF)

S1 Movie Real time imaging of lipid droplets in control *daf-2* dauer larvae in a 15-minute timeframe. (WMV)

S2 Movie Real time imaging of lipid droplets in CGI-58 mutant dauer larvae in a 15-minute timeframe. (WMV)

S3 Movie Real time imaging of lipid droplets in AMPK mutant dauer larvae in a 15-minute timeframe. (WMV)

S4 Movie Real time imaging of lipid droplets in AMPK; CGI-58 mutant dauer larvae in a 15-minute timeframe. (WMV)
