# Correction: The Causative Gene in Chanarian Dorfman Syndrome Regulates Lipid Droplet Homeostasis in *C*. *elegans*

**DOI:** 10.1371/journal.pgen.1006524

**Published:** 2016-12-21

**Authors:** Meng Xie, Richard Roy

In the title of this article, the words “Chanarian Dorfman” should read “Chanarin-Dorfman”. The correct title is: The Causative Gene in Chanarin-Dorfman Syndrome Regulates Lipid Droplet Homeostasis in *C*. *elegans*. The correct citation is: Xie M, Roy R (2015) The Causative Gene in Chanarin-Dorfman Syndrome Regulates Lipid Droplet Homeostasis in *C*. *elegans*. PLoS Genet 11(6): e1005284. doi:10.1371/journal.pgen.1005284
