# Supplementary figures and images for: The Causative Gene in Chanarian Dorfman Syndrome Regulates Lipid Droplet Homeostasis in C. elegans
Source: PLoS Genet. 2015 Jun 17;11(6):e1005284. doi: 10.1371/journal.pgen.1005284 (PMC4470697; doi:10.1371/journal.pgen.1005284)

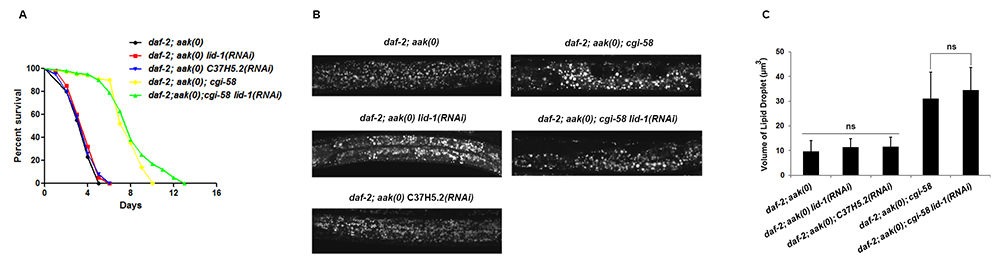

Supplement: S1 Fig — (A) lid-1 or C37H5.2 RNAi did not increase the survival of AMPK mutant dauer larvae, while lid-1 RNAi slightly increased the survival of AMPK; CGI-58 mutant dauers (P = 0.0223). (B)-(C) lid-1 or C37H5.2 RNAi did not affect the lipid droplet size in either AMPK or AMPK; CGI-58 mutant dauer larvae. (TIF) [file pgen.1005284.s001.tif]

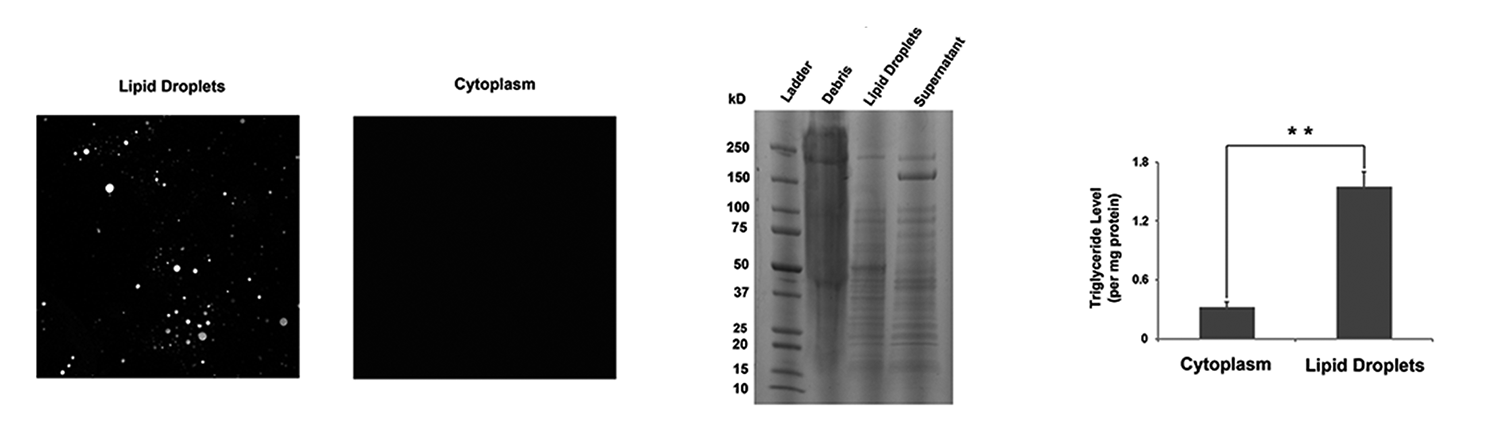

Supplement: S2 Fig — C1-BODIPY-C12 staining of isolated lipid droplets and cytoplasm (remaining portion of the total lysate) from daf-2 day 0 dauer larvae. Associated proteins present in the isolated lipid droplets were clearly distinct from that of the supernatant and the cellular debris fractions. Triglyceride was enriched several-fold in the isolated lipid droplets. (TIF) [file pgen.1005284.s002.tif]

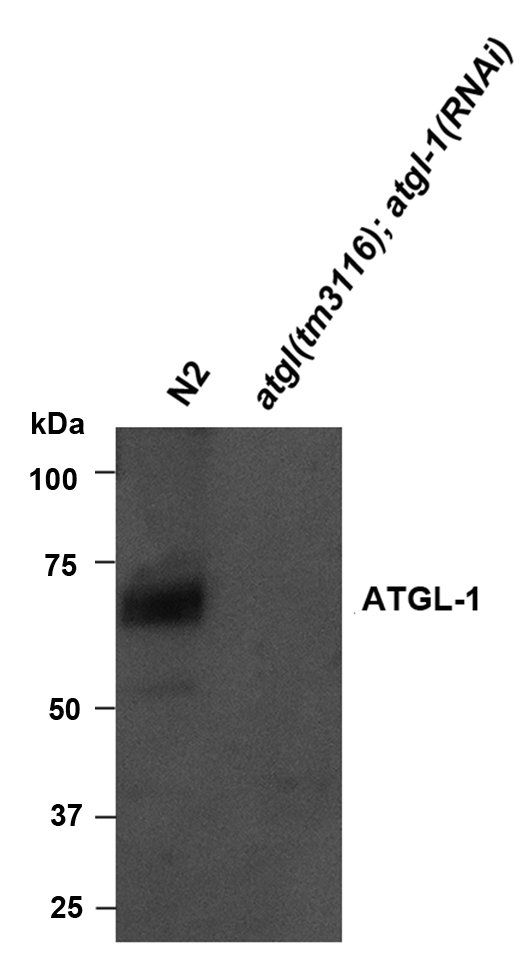

Supplement: S3 Fig — The Anti-ATGL-1 antibody is specific for ATGL-1. The ATGL-1 antisera recognizes a single band that migrates at approximately 70kD, which corresponds to the molecular weight of ATGL-1, and is reduced in atgl-1(RNAi) animals. (TIF) [file pgen.1005284.s003.tif]

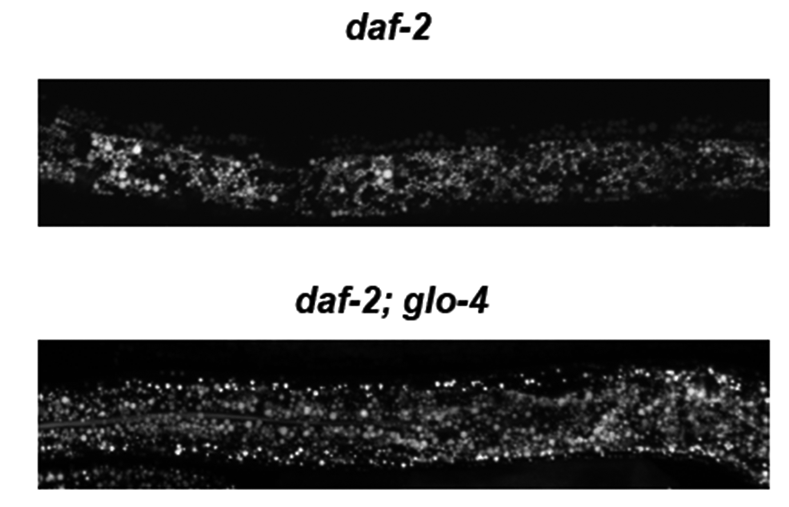

Supplement: S4 Fig — C1-BODIPY-C12 staining of lipid droplets demonstrated a similar staining pattern in daf-2 and daf-2; glo-4 (lack LROs) Day 0 Dauer Larvae, indicating that C1-BODIPY-C12 only stains lipid droplets in dauer larvae. (TIF) [file pgen.1005284.s004.tif]

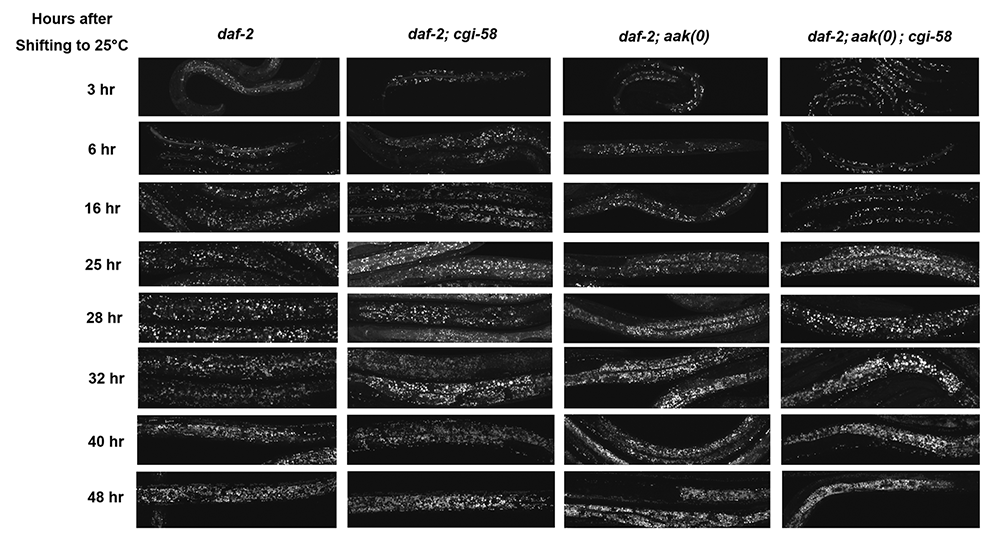

Supplement: S5 Fig — (TIF) [file pgen.1005284.s005.tif]

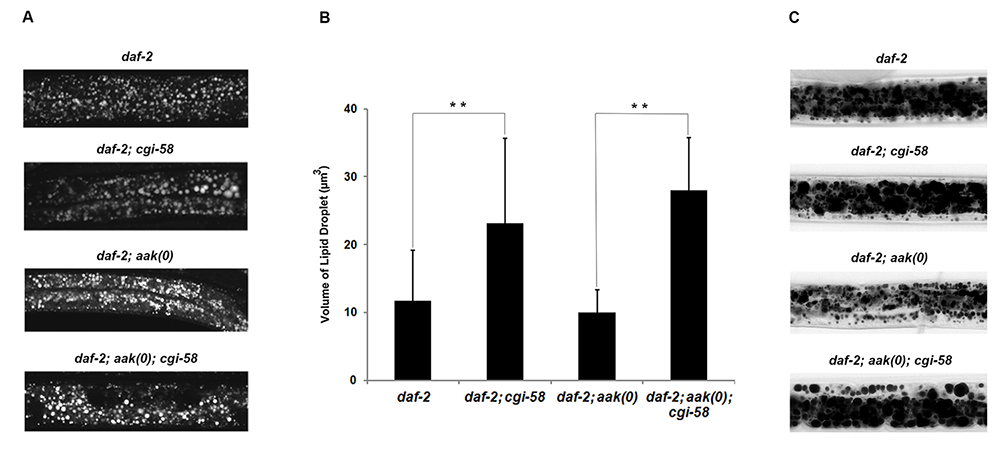

Supplement: S6 Fig — daf-2, daf-2; cgi-58, daf-2; aak(0) and daf-2; aak(0); cgi-58 Animals at the 32 hours Dauer Entry Time Point were stained either using C1-BODIPY-C12 [(A) or (B)] Oil Red O (C). No differences between the methods were observed. (TIF) [file pgen.1005284.s006.tif]

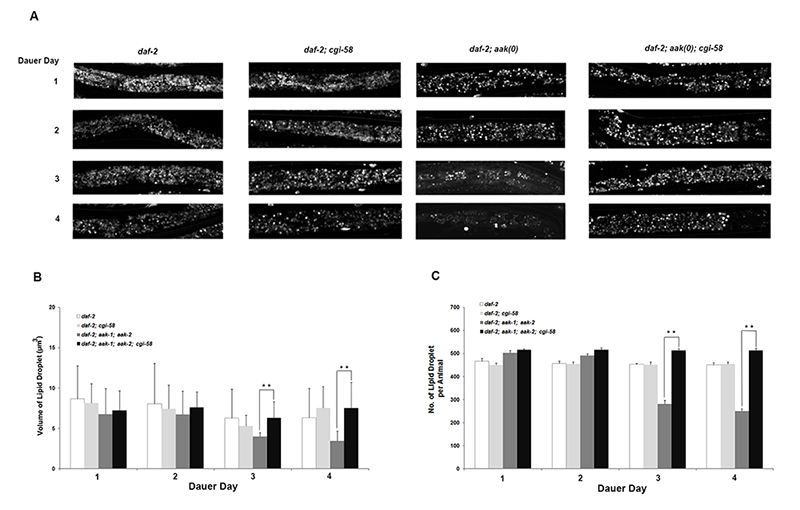

Supplement: S7 Fig — (TIF) [file pgen.1005284.s007.tif]

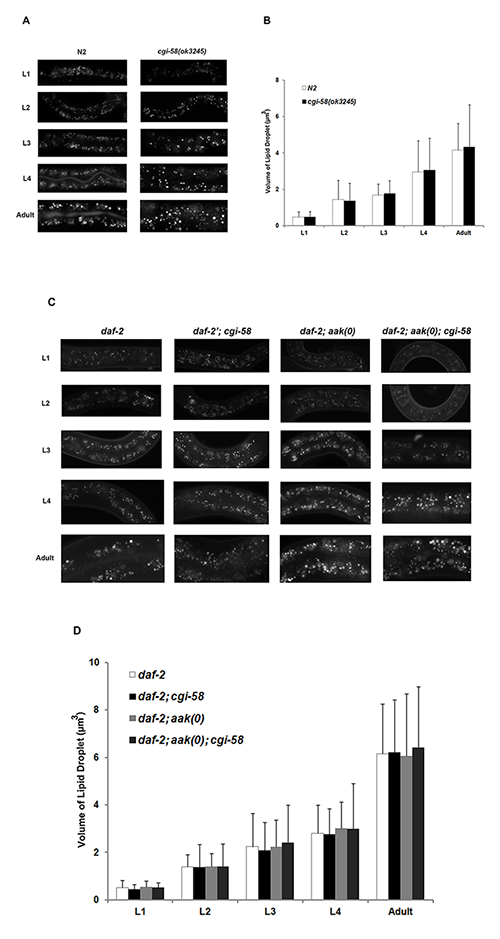

Supplement: S8 Fig — (TIF) [file pgen.1005284.s008.tif]
